# Wearable Intervention for Alcohol Use Risk and Sleep in Young Adults: A Randomized Clinical Trial

**DOI:** 10.1001/jamanetworkopen.2025.13167

**Published:** 2025-05-30

**Authors:** Lisa M. Fucito, Garrett I. Ash, Ran Wu, Brian Pittman, Nancy P. Barnett, Chiang-Shan R. Li, Nancy S. Redeker, Stephanie S. O’Malley, Kelly S. DeMartini

**Affiliations:** 1 Department of Psychiatry, Yale University School of Medicine, New Haven, Connecticut; 2 Yale Cancer Center, New Haven, Connecticut; 3 Pain, Research, Informatics, Medical Comorbidities and Education Center, Veterans Affairs Connecticut Healthcare System, West Haven, Connecticut; 4 Department of Biomedical Informatics and Data Science, Yale School of Medicine New Haven, Connecticut; 5 Department of Internal Medicine, Section of General Internal Medicine, Yale School of Medicine, New Haven, Connecticut; 6 Department of Behavioral and Social Sciences and Center for Alcohol and Addiction Studies, Brown University School of Public Health, Providence, Rhode Island; 7 Yale University School of Nursing, Orange, Connecticut; 8 School of Nursing, University of Connecticut, Storrs, Connecticut

## Abstract

**Importance:**

Young adults in the US have the highest prevalence of alcohol use disorder; technology-based interventions may help to reduce drinking.

**Objective:**

To test the efficacy of a multimodal digital intervention of wearable feedback and coaching for improving at-risk drinking and sleep health in young adults.

**Design, Setting, and Participants:**

This parallel phase 2 randomized clinical trial was conducted from December 17, 2018, to May 19, 2021, at a research clinic in Connecticut. Participants were young adults (aged 18-25 years) from the local community (web and social media ads, public flyers) with sleep concerns, 3 or more heavy drinking occasions (≥5 drinks/occasion for men; ≥4 drinks/occasion for women) in the past 2 weeks, and a positive Alcohol Use Disorders Identification Test risk score. Analyses were conducted from November 10, 2023, to September 19, 2024, using an intention-to-treat approach.

**Interventions:**

Wearable feedback and coaching plus web-based sleep advice plus smartphone self-monitoring or 1 of 2 control conditions, consisting of advice alone (control A) or advice plus self-monitoring (control A plus SM) for 2 weeks and follow-up to week 12. All participants wore sleep and alcohol biosensors.

**Main Outcomes and Measures:**

The primary outcome consisted of total number of drinks in weeks 4 to 12. Secondary outcomes included sleep disturbance, sleep-related impairment, and alcohol-related consequences in weeks 4 to 12. An exploratory outcome was also assessed, reduction of 1 or more levels in World Health Organization (WHO) drinking risk from baseline to week 4. Models compared the wearable feedback and coaching with advice and self-monitoring with each control condition and changes from weeks 4 to 8 and 12 within each condition on baseline-adjusted outcomes.

**Results:**

A total of 120 participants were enrolled (61 [51%] women; 10 [8%] Asian; 9 [8%] Black; 19 [16%] Hispanic; 96 [80%] White; 1 [1%] multiracial; 4 [3%] other race or ethnicity), with a mean (SD) age of 21.16 (1.75) years. Sixty participants were randomized to the intervention, 30 to the control A group and 30 to the control A plus SM group. Total number of drinks (primary outcome) did not differ by condition or by condition × time, but number of drinks was significantly higher at weeks 4 vs 12 (49%) across conditions. For secondary outcomes, no condition effects were observed for drinking consequences and sleep disturbance, but sleep-related impairment and WHO risk-level reduction (exploratory outcome) differed by condition. Compared with the control A group, the intervention group reported clinically meaningful lower sleep-related impairment scores (mean [SE] least square mean difference, 3.09 [1.08]; 95% CI, 0.96-5.23) and were more than 3 times more likely to have reductions in WHO risk level (odds ratio, 3.85; 95% CI, 1.34-11.07; Cohen *d* = 0.72). Sleep disturbance improvement was associated with WHO risk-level reduction.

**Conclusions and Relevance:**

This randomized clinical trial did not detect a significant effect of the intervention on the primary outcome of total drinks or secondary outcomes of sleep disturbance or drinking consequences. The intervention significantly improved other measures of sleep health and drinking reduction compared with the control A condition and warrants further testing in larger samples.

**Trial Registration:**

ClinicalTrials.gov Identifier: NCT03658954

## Key Points

**Question:**

Is a wearable feedback and coaching intervention efficacious for improving at-risk drinking and sleep health in young adults?

**Findings:**

In this phase 2 randomized clinical trial including 120 young adults, a 2-session multimodal sleep-alcohol wearable feedback and coaching intervention did not significantly reduce total drinks compared with web-based advice alone or with smartphone self-monitoring. All conditions had clinically meaningful drinking reductions over time, but the intervention showed a benefit over advice alone for other sleep health and drinking reduction outcomes.

**Meaning:**

These findings suggest that further research is warranted to optimize the intervention and evaluate its efficacy for alcohol risk reduction in young adults.

## Teaser

This randomized clinical trial compared an intervention with a core component of feedback and coaching plus web-based advice plus smartphone diary self-monitoring with 2 control conditions among young adults with sleep concerns and risk for alcohol misuse.

## Introduction

Young adults are a priority population for alcohol-related prevention and intervention strategies, as they are disproportionately affected by alcohol use.^1^ Digital health technologies might offer solutions. Young adults increasingly use digital mental and physical health tools, including wearable devices (wearables)^2^ for their anonymity, privacy, accessibility, inclusivity, sense of community, and self-direction.^3^ Various technological modalities (eg, computer-based or web programs, applications, video games) have been used to reduce young adult substance use.^4,5,6,7^ Most interventions provided alcohol education and brief advice, included personalized drinking feedback and normative comparisons with same-age peers, and demonstrated small and/or inconsistent effects.^4,5,6,7^

Notably, alcohol interventions integrating wearables warrant investigation, given their potential for behavior change.^8^ For physical health, wearables have been investigated for monitoring, predicting health status, phenotyping, and promoting behavior change.^8,9^ Their application to behavioral health, such as alcohol use, is nascent.^10,11^ Alcohol sensor technology can detect transdermal alcohol concentration from sweat.^12^ Research on a continuous alcohol monitor (SCRAM CAM; SCRAM Systems), the most studied device, has focused on binary drinking detection primarily in criminal justice populations.^13^ New wrist-worn alcohol wearables resemble fitness wearables and offer less burdensome assessment.^13^ Despite the availability and validity of alcohol detection wearables, their efficacy for reducing drinking is uncertain.^11^

Whether fitness and sleep wearables, alone or combined with alcohol wearables, reduce drinking has not to our knowledge been tested.^14^ Interventions that integrate multiple health domains, such as sleep, align with young adults’ preferences^15^ and increase engagement in those ambivalent about changing their drinking.^16^ Consistent with behavior change theories, key health behavior change mechanisms include increasing awareness of health behavior risks and benefits, promoting motivation and self-efficacy, reducing barriers, and providing practical behavioral skills.^17,18^ Thus, an alcohol intervention that leverages personalized wearable feedback to explicitly link drinking to other health biometrics and enhances confidence and goal achievement with tailored coaching could be effective.

For young adults, targeting the association between drinking and sleep using wearables merits further study. Disturbances in sleep and circadian rhythms are associated with drinking and alcohol-related problems.^19^ These dual risks often cluster in young people.^20^ Mechanistically, sleep and circadian factors may contribute to alcohol risk by enhancing alcohol’s rewarding effects, impairing mood and emotion regulation, and/or interacting with neurobiological craving and impulsivity pathways.^19^ Therefore, improved sleep and circadian health might reduce drinking through changes in these pathways. Moreover, young adults who report at-risk drinking are open to sleep interventions^21^; preliminary evidence finds that these treatments may engage them in learning about their alcohol use and reduce sleep and alcohol-related problems.^22,23^ Further study is needed to understand whether sleep interventions reduce young adult drinking. To meet this need, we developed a brief multimodal digital intervention with personalized sleep and alcohol wearable feedback and tailored virtual coaching for alcohol risk reduction in young adults (Call It a Night), which was informed by rigorous formative research,^21^ behavior change theory, evidence-based frameworks^24^ and interventions,^25^ and pilot testing.^23^

## Methods

### Design

This study was a single-site, 3-condition, randomized clinical trial (RCT) in young adults (N = 120) to identify efficacious digital intervention components for inclusion in a larger-scale efficacy trial of the multimodal intervention vs standard care. Based on the pilot trial including some of our investigators,^23^ we compared the intervention against 2 control conditions that did not include the core component of feedback and coaching but varied in other components: (1) web-based advice only (control A) or (2) web-based advice plus smartphone diary self-monitoring (control A plus SM). The 2-control condition design allowed evaluation of feedback and coaching relative to the other active treatment components. The Yale University Institutional Review Board approved the protocol (Supplement 1). The methods described herein follow the Consolidated Standards of Reporting Trials (CONSORT) guideline for RCTs. All participants provided written informed consent.

### Participants

**Figure 1.**
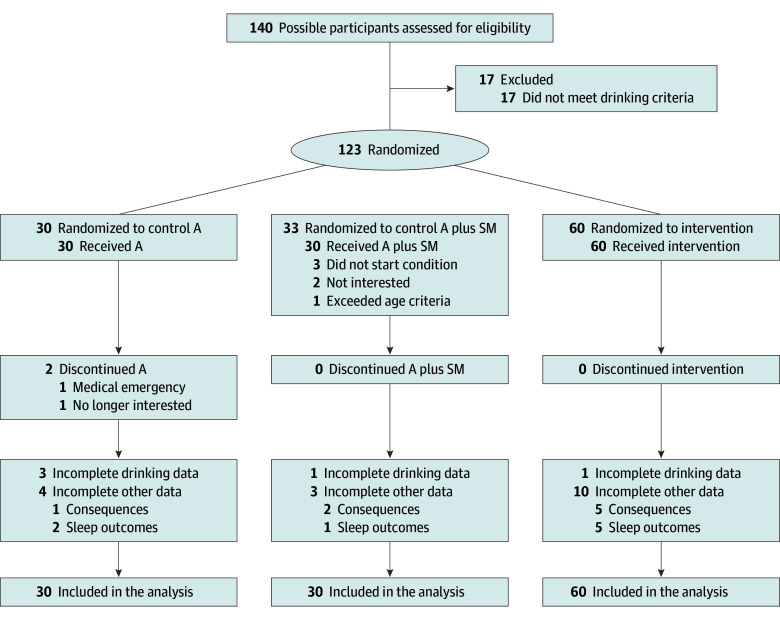
CONSORT Flow Diagram A indicates advice; A plus SM, advice plus self-monitoring.

Key inclusions were 18 to 25 years of age, reporting 3 or more heavy drinking occasions in the past 2 weeks (ie, ≥5 drinks/occasion for men; ≥4 drinks/occasion for women), and endorsing at-risk drinking with a positive Alcohol Use Disorders Identification Test risk score^26^ and sleep concerns. Key exclusions were self-reported current alcohol or sleep treatment, sleep disorder history (eg, sleep apnea), severe alcohol withdrawal or psychiatric illness, or substance use disorder (excluding cannabis) based on diagnostic interview.^27^ More details are found in the protocol (Supplement 1), and a separate version has been published elsewhere.^25^ The participant flow diagram appears as Figure 1.

### Randomization and Masking

Participants were randomly assigned 2:1:1 to (1) the intervention consisting of 2 sessions of feedback plus coaching (n = 60), (2) control A (n = 30), or (3) control A plus SM (n = 30). Randomization, stratified by sex, occurred through a web-based system to conceal allocation. An independent statistician (R.W.) generated the randomization sequence and conducted analyses blind to condition. Staff and participants were not blind due to the nature of the interventions. The analysis plan was developed a priori by a statistician except for the World Health Organization (WHO) drinking risk-level reduction analysis, which was added a posteriori based on research published after trial initiation on its utility as a clinical end point^28,29,30,31,32,33,34,35,36,37,38,39^ and for alcohol risk phenotyping.^37^

A sample of 120 participants was estimated to detect a clinically meaningful medium effect for the primary outcome of total drinks, controlling for baseline, based on the pilot study results^23^ under these assumptions: (1) significance threshold of 2-sided α = .05, (2) 80% power, (3) medium effect (Cohen *f* = 0.29), (4) 1 binary stratification variable (sex), and (5) 10% dropout. For post hoc tests comparing the intervention with each control (A and A plus SM), we were powered to detect medium effects (Cohen *d* = 0.63).

### Procedures

The study was conducted from December 17, 2018, to May 19, 2021, at a Connecticut research clinic. Volunteers, recruited through online and social media advertising and flyers posted in the community, attended an intake to provide written informed consent and complete assessments. Eligible participants were randomized, received interventions during 2 weeks, completed follow-ups at weeks 4, 8, and 12, and were compensated.

#### Wearable Passive Self-Monitoring

During the 2-week intervention, all participants wore a sleep biosensor (Actiwatch Spectrum Plus; Philips Respironics) and an alcohol biosensor (SCRAM CAM; SCRAM Systems). Neither wearable provided feedback. Sleep actigraphy is valid, reliable, and sensitive to intervention effects.^40^ The continuous alcohol monitoring device is valid and reliable for detecting drinking by sampling transdermal alcohol concentrations from sweat.^12^

#### Web-Based Sleep Advice

All participants viewed a 2-module interactive web-based sleep program^21,23^ during brief midweek research appointments at weeks 1 and 2. The program focused on healthy sleep (7-9 hours, sleep hygiene, and regular sleep timing)^41^ and included evidence-based alcohol intervention content^42^ (Table 1).

**Table 1.** Intervention Components by Condition

Intervention component	Intervention condition		
	Control A	Control A plus SM	Intervention
**2-Module web-based brief sleep health advice**			
Module 1 (10 min)			
Basic sleep overview	Yes	Yes	Yes
Behaviors that affect sleep including alcohol use	Yes	Yes	Yes
General behavioral recommendations for optimal sleep	Yes	Yes	Yes
How to establish a good sleep routine/schedule, strengthen bed/bedroom as sleep cues	Yes	Yes	Yes
Basic overview of alcohol effects, standard drink conventions(, blood alcohol levels including the maximum level (0.06%) to minimize negative effects	Yes	Yes	Yes
Low-risk drinking guidelines	Yes	Yes	Yes
Norms-based drinking comparisons with same-sex peers	Yes	Yes	Yes
Advice to moderate drinking to reduce harm	Yes	Yes	Yes
Alcohol effects on sleep, potential negative synergistic effects of deficient sleep and alcohol use	Yes	Yes	Yes
Module 2 (10 min)			
Optimal internal and external sleep factors to promote sleep, synchronize circadian rhythm, and reduce sleep-interfering arousal or activation	Yes	Yes	Yes
3 Brief video clips demonstrating deep breathing, progressive muscle relaxation, mindfulness meditation skills	Yes	Yes	Yes
**Daily smartphone diaries**			
Sleep			
Bed and wake time	No	Yes	Yes
Total No. of awakenings and reasons for waking	No	Yes	Yes
Self-reported sleep quality, mood, sleepiness, and alertness upon final waking	No	Yes	Yes
Alcohol			
Total number of standard drinks consumed, start and stop time of drinking occasions	No	Yes	Yes
Drinking consequences	No	Yes	Yes
Cravings to drink	No	Yes	Yes
Drinking context (alone vs with others, public vs private place)	No	Yes	Yes
Other substance use	No	Yes	Yes
**2 Sessions of personalized integrated wearable feedback and tailored coaching**			
Visual displays of wearable & daily diary data via electronic handouts	No	No	Yes
Review of health guidelines for sleep & alcohol use among young adults	No	No	Yes
Personalized sleep and alcohol use associations as indicated by wearable and diary data	No	No	Yes
Setting, implementing, and sustaining sleep and alcohol use behavioral goals	No	No	Yes
Sleep and alcohol use behavior change strategies (eg, drinking to lower BAC, stopping alcohol use earlier, shifting bedtime earlier, getting morning bright light exposure)	No	No	Yes

Abbreviations: A, advice; A plus SM, advice plus self-monitoring; BAC, blood alcohol concentration.

#### Smartphone Self-Monitoring

Participants assigned to the intervention and A plus SM monitored their sleep and drinking daily for 14 days via smartphone diaries.^25^ Details are found in Table 1 and eFigure 1 in Supplement 2.

#### Intervention

Intervention participants received personalized feedback based on their wearable and smartphone diary data and tips tailored to these data in 2 virtual sessions with a health coach at the end of weeks 1 and 2 (Table 1). Two PhD-level coaches with lifestyle health expertise delivered the manualized sessions (G.I.A., kinesiologist [n = 17]; L.M.F, psychologist [n = 35]; or both [n = 8]).

Electronic handouts summarized sleep and alcohol biosensors and daily diary data. Visualizations showed associations among sleep, alcohol use, and substance use (eFigure 2 in Supplement 2). Estimated blood-alcohol concentrations (BAC) from diaries were overlaid on daily sleep and wake data for drinking near bedtime. Sleep periods were superimposed over alcohol biosensor–detected transdermal alcohol concentrations and BAC curves from daily diaries, highlighting alcohol metabolism duration, BAC elevations during sleep and waking, and differences in sleep on drinking vs nondrinking occasions.

Using a motivational enhancement approach, the coach reviewed feedback reports with participants, emphasizing autonomy to change.^25^ Coaches explained health variables and the derivation of composite data and conducted open-ended goal discussions. Coaches affirmed positive health behaviors, communicated clear advice to change risky behaviors, and highlighted sleep-alcohol associations. Participants were offered tailored behavioral strategies (Table 1). Session 1 focused on strategy experimentation and session 2 on strategy evaluation and long-term goals.

### Measures

Participants self-reported sex at birth, race and ethnicity, and educational attainment at baseline. Self-reported race included Asian, Black, White, multiracial, and other (ie, not specified); ethnicity was self-reported as Hispanic or non-Hispanic. These data provide information that may be important for the generalizability of study findings. The Timeline Follow-back Interview^43^ assessed past 14-day total drinks at intake and follow-up. We converted the interview data into WHO risk level for drinking^31,37^ for each time point: very high (men: >100 g/d; women: >60 g/d), high (men: 60-100 g/d; women: 40-60 g/d), moderate (men: 40-60 g/d; women: 20-40 g/d), low (men: 1-40 g/d; women: 1-20 g/d), and abstinence. We coded data for a reduction of 1 or more levels in WHO risk from baseline to each follow-up. A reduction of 1 or more levels was prioritized since nearly one-quarter of participants had a low risk level at baseline, eliminating the possibility of a reduction of 2 or more levels. WHO risk-level reduction, a clinically meaningful alcohol intervention end point,^28,30,32,44,45^ is associated with improvements in sleep, psychological functioning, quality of life, and health care costs.^31,34,35,37,38,39^ At baseline and follow-up, the Brief Young Adult Alcohol Consequences Questionnaire^46^ assessed drinking-related problems, and the 8-item Patient Reported Outcomes Measurement Information System (PROMIS) Sleep Disturbance and Sleep-Related Impairment Short Forms assessed sleep health.

### Statistical Analyses

Data were analyzed from November 10, 2023, to September 19, 2024. We used an intention-to-treat approach for the 120 participants who started treatment. Data were evaluated for skewness and kurtosis and transformed as needed. Analyses were conducted with SAS, version 9.4 (SAS Institute Inc). The primary alcohol outcome was total number of standard drinks (1 standard drink = 14 g), calculated as a mean during 14 days at each time point. Secondary outcomes were sleep disturbance, sleep-related impairment ratings, and alcohol-related consequences. A reduction of 1 or more levels in WHO risk level from baseline (1 = yes; 0 = no) was an exploratory outcome.

Linear mixed models examined condition effects on primary and secondary outcomes, using all available data. Fixed effects included condition, time (weeks 4, 8, and 12), and their interaction, controlling for baseline values and sex. Random participant effects were modeled, with compound symmetry covariance structure fitting most outcomes except consequences, which used a first-order autoregressive structure according to information criteria. Total drinks and consequences, being nonnormally distributed, were log and square root transformed, respectively.

Planned post hoc comparisons of least square mean differences (LSMD) for both primary and secondary outcomes included (1) intervention vs each control (A and A plus SM) calculated as the mean over time (between participants) and (2) changes from week 4 at weeks 8 and 12 for each condition (within participants). Although combining the 2 controls would increase power, per protocol analyses were not combined, providing greater treatment effects insights. Total number of drinks (primary outcome) was tested at the 2-sided .05 α level. For secondary outcomes, we used Bonferroni correction to adjust post hoc contrasts for multiple comparisons. Specifically, the 2 between-participants contrasts were tested at an adjusted α of .025 (0.05/2), while the 6 within-participant comparisons were tested at an adjusted α of .008 (0.05/6). These thresholds were chosen based on a priori planned comparisons while avoiding being unduly conservative.

The analysis of WHO risk-level reduction from baseline was limited to week 4 because more than 50% of participants reached low-risk or abstinence levels by week 4, making further reductions unlikely, and to best assess the largest drinking reduction between baseline and week 4 (Table 2 and Table 3). This strategy is consistent with prior research.^28^ Missing data for 4 participants were conservatively coded as no reduction. Logistic regression was used to model the likelihood of achieving a reduction of 1 or more levels from baseline in WHO risk level (coded as 1 = ≥1 level reduction; 0 = no reduction) at week 4. The model included condition and controlled for sex and baseline WHO risk level. Post hoc analyses were tested at a 2-sided α = .025, adjusting for the 2 comparisons of the intervention with each control (A and A plus SM). We performed a sensitivity analysis excluding the 4 participants with missing outcome data.

**Table 2.** Baseline Demographic and Clinical Characteristics of Participants by Condition (N = 120)

Characteristic	Condition, participants, No. (%)^a^		
	Control A (n = 30)	Control A plus SM (n = 30)	Intervention (n = 60)
Gender			
Men	15 (50)	14 (47)	30 (50)
Women	15 (50)	16 (53)	30 (50)
Age, mean (SD), y	20.93 (1.72)	21.3 (1.84)	21.2 (1.74)
Race			
Asian	3 (10)	2 (7)	5 (8)
Black	1 (3)	1 (3)	7 (12)
Multiracial	0	0	1 (2)
Other^b^	0	2 (7)	2 (3)
White	26 (87)	25 (83)	45 (75)
Ethnicity			
Hispanic	5 (17)	5 (17)	9 (15)
Non-Hispanic	25 (83)	25 (83)	51 (85)
Student	22 (73)	23 (77)	43 (72)
Total No. of drinks past 2 wk, mean (SD)	39.20 (18.17)	49.02 (23.85)	45.16 (22.68)
WHO drinking risk level^c^			
Low	11 (37)	5 (17)	13 (22)
Medium	13 (43)	12 (40)	28 (47)
High	4 (13)	10 (33)	16 (27)
Very high	2 (7)	3 (10)	3 (5)
PROMIS Sleep Disturbance score, mean (SD)^d^	53.40 (4.83)	55.26 (5.04)	53.38 (5.48)
PROMIS Sleep-Related Impairment score, mean (SD)^d^	58.09 (9.04)	58.26 (6.86)	56.86 (6.56)

Abbreviations: A, advice; A plus SM, advice plus self-monitoring; PROMIS, Patient Reported Outcomes Measurement Information System Short Forms; WHO, World Health Organization.

^a^ Control A received web-based sleep advice only; control A plus SM, web-based sleep advice plus smartphone self-monitoring; and intervention, personalized wearable feedback plus tailored coaching plus A plus SM.

^b^ Participants endorsed other but did not specify a response.

^c^ Defined as very high (men: >100 g/d; women: >60 g/d), high (men: >60-100 g/d; women: >40-60 g/d), moderate (men: >40-60 g/d; women: >20-40 g/d), and low (men: 1-40 g/d; women: 1-20 g/d).

^d^ Scores range from 0 to 100; higher scores indicate worse sleep health outcomes.

**Table 3.** Descriptive Statistics for Treatment Outcomes by Condition (N = 120)

Outcome	Mean (SD)				Model	*P* value
	Baseline	Week 4	Week 8	Week 12		
**Primary: total No. of drinks past 2 wk^a^**						
Control A	39.20 (18.17)	31.07 (22.84)	22.04 (15.14)	22.16 (17.29)	Condition: F_2, 112_ = 0.26	.77
Control A plus SM	49.02 (23.85)	35.16 (24.20)	29.77 (22.96)	23.94 (22.92)	Time: F_2, 226_ = 8.63	<.001
Intervention	45.16 (22.68)	30.53 (20.20)	29.29 (29.67)	27.51 (26.13)	Condition × time: F_4, 226_ = 0.78	.54
**Primary: PROMIS Sleep Disturbance Short Form score^b^**						
Control A	53.40 (4.83)	51.63 (4.52)	50.65 (5.70)	51.12 (5.47)	Condition: F_2, 111_ = 2.72	.07
Control A plus SM	55.26 (5.04)	51.31 (5.59)	50.18 (4.09)	49.83 (5.71)	Time: F_2, 221_ = 0.97	.38
Intervention	53.38 (5.48)	49.19 (5.56)	49.23 (4.91)	49.36 (4.75)	Condition × time: F_4, 221_ = 0.65	.63
**Secondary: alcohol-related consequences^a^**						
Control A	9.23 (4.97)	8.34 (5.09)	4.93 (4.46)	4.93 (4.46)	Condition: F_2, 114_ = 0.66	.52
Control A plus SM	11.43 (5.18)	7.64 (4.21)	7.57 (6.49)	7.57 (6.49)	Time: F_2, 217_ = 14.30	<.001
Intervention	9.47 (5.78)	6.95 (5.55)	5.64 (4.57)	5.64 (4.57)	Condition × time: F_4, 217_ = 3.95	.004
**Secondary: PROMIS Sleep-Related Impairment Short Form score**						
Control A	58.09 (9.04)	58.15 (6.41)	57.26 (7.41)	58.46 (7.42)	Condition: F_2, 110_ = 4.86	.01
Control A plus SM	58.26 (6.86)	58.20 (7.78)	57.19 (4.44)	56.69 (7.98)	Time: F_2, 221_ = 1.42	.25
Intervention	56.86 (6.56)	55.60 (6.03)	54.73 (6.55)	53.66 (7.16)	Condition × time: F_4, 221_ = 0.58	.68

Abbreviations: A, advice; A plus SM, advice plus self-monitoring; PROMIS, Patient Reported Outcomes Measurement Information System Short Forms.

^a^ Indicates an outcome that was transformed for analysis although nontransformed data reported in Table (total drinks – log transformed; consequences – sqrt transformed).

^b^ Scores range from 0 to 100; higher scores indicate worse sleep outcomes.

## Results

### Participants

A total of 120 young adults were enrolled between February 13, 2019, and February 24, 2021. Baseline characteristics by condition are shown in Table 2. A total of 59 men (49%) and 61 women (51%) had a mean (SD) age of 21.16 (1.75) years. Ten participants (8%) were Asian; 9 (8%), Black; 96 (80%), White; 1 (1%), multiracial; and 4 (3%), other race or ethnicity. Nineteen participants (16%) self-reported Hispanic ethnicity.

### Feasibility

Across conditions, adherence to daily diary completion (114 [95%]) and daily wearable use (ie, 14 days’ worth of analyzable data) was high (sleep, 117 [98%]; alcohol, 114 [95%]). Nearly all participants completed treatment and follow-up (Figure 1).

### Primary Outcome

#### Total Drinks

There was a significant time effect but no condition effect or condition × time interaction (Table 2). Total number of drinks (log) decreased significantly between weeks 4 and 8 (LSMD [SE], 0.27 [0.10]; 95% CI, 0.08-0.47; *t*_226_ = 2.77; *P* = .006) and between weeks 4 and 12 (LSMD [SE], 0.40 [0.10]; 95% CI, 0.21-0.60; *t*_227_ = 4.06; *P* < .001). Effect sizes were computed by back-transforming LSMDs to their original scale, which is equivalent to the adjusted geometric mean ratio (GMR). Total number of drinks at week 4 were 31% (GMR, 1.31) and 49% (GMR, 1.49) times higher than those observed at weeks 8 and 12, respectively.

### Secondary Outcomes 

#### Drinking Consequences

There were significant time effects and a condition × time interaction on Brief Young Adult Alcohol Consequences Questionnaire score (transformed using square root). Specifically, drinking consequences decreased over time for participants in the intervention and control A but not control A plus SM groups. Among control A participants, compared with week 4, consequences were lower at week 8 (LSMD [SE], 0.87 [0.15]; 95% CI, 0.58-1.15; *t_215_* = 5.91; *P* < .001) and week 12 (LSMD [SE], 0.87 [0.19]; 95% CI, 0.49-1.25; *t_269_* = 4.49; *P* < .001), with both significant at the adjusted α = .008 threshold. Among the intervention participants, compared with week 4, consequences were lower at week 8 (LSMD [SE], 0.22 [0.11]; 95% CI, 0.01-0.43; *t*_218_ = 2.06; *P* = .04) but not at week 12 (LSMD [SE], 0.24 [0.14]; 95% CI, −0.19 to 0.23; *t*_272_ = 1.70; *P* = .09). However, the observed change at week 8 was not significant at the adjusted α = .008 threshold.

#### Sleep Disturbance 

Although the condition effect did not reach significance and the time and interaction effects were not significant, preplanned contrasts indicated that intervention participants had significantly lower sleep disturbance scores calculated as the mean over time compared with control A participants (LSMD [SE], 1.83 [0.80];95% CI, 0.25-3.40; *t*_111_ = 2.29; *P* = .02), which was significant at the adjusted α = .025 threshold. However, the intervention and A plus SM control groups did not differ.

#### Sleep-Related Daytime Impairment

There was a significant condition effect on sleep-related daytime impairment but no time effect or condition × time interaction. With means calculated over time, intervention participants had significantly lower sleep-related impairment scores compared with both the control A plus SM (LSMD [SE], 2.22 [1.07]; 95% CI, 0.11-4.34; *t*_111_ = 2.08; *P* = .04) and control A (LSMD [SE], 3.09 [.08];95% CI, 0.96-5.23; *t*_110_ = 2.88;, *P* = .01) groups. However, only the comparison between the intervention and control A groups was significant at the adjusted α = .025 level.

### Exploratory Outcome and Potential Associations

#### WHO Drinking Risk-Level Reduction

**Figure 2.**
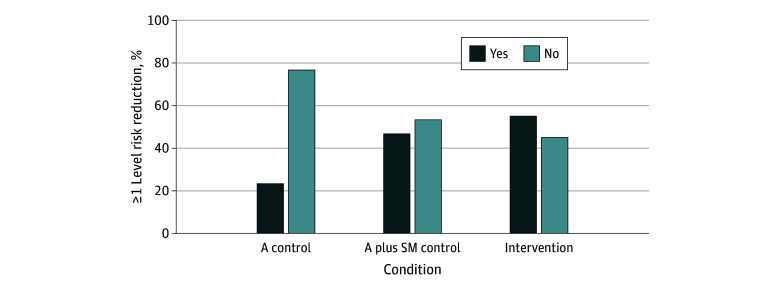
Percentages of Participants Who Achieved and Did Not Achieve Reduction of 1 or More Levels of World Health Organization Risk A indicates advice; A plus SM, advice plus self-monitoring.

The proportion of individuals with a reduction of 1 or more levels in WHO drinking risk from baseline to week 4 differed significantly by condition (Figure 2). Specifically, intervention participants were more likely to reduce their risk by 1 or more WHO risk level than control A participants (odds ratio [OR], 3.85; 95% CI, 1.34-11.07; *P* = .01; Cohen *d* = 0.72), which was significant at the adjusted α = .025 level. The sensitivity results were comparable (OR, 3.19; 95% CI, 1.09-9.29; *P* = .03). The intervention and control A plus SM conditions did not differ.

#### Associations Among WHO Risk-Level Reduction, Sleep Improvement, and Condition

We explored potential sleep-alcohol associations by deriving change scores from baseline to week 4 for both PROMIS sleep outcomes and evaluating them as variables to estimate week 4 reduction of WHO risk-level status. Reduction in sleep disturbance was associated with WHO risk-level reduction by week 4 (χ^2^_1_ = 5.07; *P* = .02). Specifically, those who experienced greater reduction in sleep disturbance were more likely to achieve a reduction of 1 or more levels in WHO risk (OR, 1.11; 95% CI, 1.02-1.20). When this sleep disturbance change score was added to the main model estimating WHO risk-level reduction at week 4, the condition effect was not significant (χ^2^_2_ = 4.21; *P* = .12). Change in sleep impairment was not associated with week 4 WHO risk-level reduction.

## Discussion

This RCT examined the effect of a multimodal digital intervention incorporating wearable feedback and coaching for improving at-risk drinking and sleep health in young adults. The intervention did not show a significant benefit for the primary outcome of total number of drinks or secondary outcomes of consequences compared with active controls. Participants in all conditions significantly reduced their drinking and alcohol consequences. Mean total number of drinks was significantly higher at weeks 4 (49%) and 8 (31%) than week 12. While post hoc tests showed that the intervention yielded significantly lower secondary sleep disturbance scores than the control A condition, the sample size was powered to detect clinically meaningful medium effects, not the smaller effect observed for this outcome. However, the intervention resulted in significantly greater and clinically meaningful improvements on the secondary outcome of sleep-related daytime impairment and a medium effect size for the exploratory outcome of WHO drinking risk-level reduction compared with the control A condition. Our exploratory analyses suggest improved sleep health was associated with likelihood of achieving a WHO drinking risk-level reduction.

Importantly, the results demonstrated high feasibility and acceptability for this integrated intervention approach. Across all conditions, retention and adherence were very high. Published analysis of exit survey and interview data showed high satisfaction across conditions and that sleep, not drinking, was the primary motivation for participation.^47^ The intervention was rated as most effective; participants preferred feedback and coaching over advice and self-monitoring.^47^

The lack of significant difference for the intervention participants compared with controls on the primary drinking outcome and some secondary outcomes may be due to several factors. All participants received active appealing interventions^47^ that included evidence-based alcohol^42^ and sleep content.^41^ Participant reactivity to repeated alcohol assessments,^48^ especially daily self-monitoring in the control A plus SM and intervention conditions, could also be a factor. Whereas motivation to change alcohol use was low in this sample, the intervention may have greater efficacy among young adults actively seeking alcohol treatment. In addition, there was no sleep-related clinical threshold for enrollment, although the intervention’s effect on sleep-related impairment (approximately a 3-point reduction) was within the suggested minimum clinically important difference for PROMIS sleep outcomes.^49^

The intervention shows potential as a scalable strategy for engaging young adults and improving their drinking and sleep health^19^ and could be modified to address cost, technology, and other barriers. Wearable devices are now available in low-cost simple models, and there are free smartphone applications for tracking sleep, drinking, and other behaviors. Coaching and/or self-monitoring can be implemented via SMS (short message service) text-messaging, and written materials might replace web-based advice. Health care professionals could advise patients to track sleep and fitness and explore potential connections with drinking at follow-up. Nevertheless, it is critical that these digital tools be rigorously investigated, accounting for various factors, including dosage, components, and coach’s training, to inform best implementation practices.

### Strengths and Limitations

Study strengths include the novelty of repurposing alcohol wearables from a legal monitoring device into a digital therapeutic device^50^ and the integration of multimodal wearable and smartphone diary data with coaching. The study was scientifically rigorous and used a theoretical foundation in multiple health behavior change, a user-centered design that informed the intervention, and RCT design. The study had very low missing data (approximately 4% for the primary outcome) and limited exclusion criteria, and it incorporated a manual and televideo for coaching and a standardized feedback report template.

This study also has some limitations. These include a single US site, a mostly non-Hispanic White student sample, subjective outcome assessment, blinding of the statistician only, the a posteriori WHO outcome, brief treatment and follow-up, limited power to detect less than medium effects, and only accelerometer data included in the sleep wearable.

## Conclusions

Although all digital intervention components had appeal and utility for reducing drinking, in this RCT, the intervention, which included wearable feedback and coaching, showed the most promise for clinically meaningful improvements in sleep health and alcohol risk reduction. A larger fully powered trial is needed in more diverse populations, under clinical conditions, and with a longer duration to assess efficacy, scalability, and sustainability. These findings have implications. Interactive digital health technologies that address alcohol use within a transdiagnostic, holistic framework may be particularly appealing for young adults.^16,47^ The findings have relevance to other health domains and personalized feedback and coaching interventions that integrate multiple wearable data.^51,52^ Visual presentations and artificial intelligence–derived tools that can help users connect the dots across health behaviors and biometrics are exciting and underexplored opportunities of digital health technologies.

## Notes

Supplement 1.
Trial Protocol

Supplement 2. **eFigure 1.** Sample Smartphone Diary Image **eFigure 2.** Sample Participant Feedback

Supplement 3.
Data Sharing Statement
