# Supplementary material for: Wearable Intervention for Alcohol Use Risk and Sleep in Young Adults: A Randomized Clinical Trial
Source: JAMA Netw Open. 2025 May 30;8(5):e2513167. doi: 10.1001/jamanetworkopen.2025.13167 (PMC12125640; doi:10.1001/jamanetworkopen.2025.13167)
Supplement: Supplement 1. — Trial Protocol [file jamanetwopen-e2513167-s001.pdf]

Table of Contents

I. Summary of Changes to the Protocol…………………………2.

II. Original Protocol at Trial Initiation …………………………2.

I. Summary of Changes to the Protocol

Amendments to the Protocol	Approval Date
Added self-report questionnaires to evaluate the potential theoretical mechanisms of the intervention effects; modified the number of times the Go/No Go task was to be administered to at baseline and week 4; modified the drinking inclusion criteria to be at least 3 occasions of heavy drinking in the last 2 weeks; updated the statistical analysis section by providing more detail about approach; modified the Fitbit device to a Fitbit Charge 2; updated procedures for managing the use of wearable technology including security concerns and adherence.	12/21/17
Replaced the Go/No Go task with a Stop Signal task and N-back task, revised the alcohol weararble from the Milo wrist-worn sensor to the ankle-worn, SCRAM. Removed urine drug screen at follow-up weeks. Added height, weight and physical fitness survey at intake. Updated smartphone diary questions. Changed self-reported sleep to PROMIS and mood measures to PANAS. Changed Actiwatch for Fitbit to measure objective sleep outcomes. Revised timing of behavioral mechanism assessments. Updated community-based recruitment methods and updated compensation schedule to optimize retention.	12/17/18
Proposed to add mobile psychomotor vigilance testing (PVT) as an additional sleep outcome, add added new recruitment ads.	3/26/19
Updated and increased compensation.	6/20/19
Revised study to replace the Milo Proof write-worn alcohol biosensor with the BACtrack Skyn.	7/26/19
Added new study ads.	9/27/19
Revised consent form language to add a new notice and signature point for potential enrolled participants indicating that they accept responsibility for the devices and research equipment.	10/5/19
Added new study ads.	1/13/20

II. Original Protocol at Trial Initiation (Yale Site)

Title of Research Project: *Development of a Multimodal Sleep Intervention Using Wearable Technology to Reduce Heavy Drinking in Young Adults*

Principal Investigator: Lisa Fucito, Ph.D.

Date: 7/02/17

Probable Duration of Project: We anticipate that this project will take approximately 3 years to complete and analyze.

Targeted Enrollment: The targeted enrollment for this protocol is 120 participants plus 5 pilot participants who will not be included in our statistical analyses.

SECTION I: RESEARCH PLAN

1. **Statement of Purpose:** State the scientific aim(s) of the study, or the hypotheses to be tested.

An important correlate and potential consequence of poor sleep is AUD (Alcohol Use Disorder) risk. In young adults, greater alcohol consumption and alcohol-related consequences are associated with shorter sleep duration, poorer sleep quality, and more delayed bed/wake times.[10, 11] In addition, sleep problems in adolescence (i.e., short sleep duration, difficulty falling/staying asleep, and variable sleep timing) predict greater risk of an AUD, earlier AUD onset, and greater risk of heavy-drinking and alcohol-related consequences in young adulthood.[12-14] Further, poor sleep in young adults predicts greater future risk of alcohol-related consequences.[20]

The mechanism that accounts for these sleep-alcohol associations in young adults is not clear. One possible theory is that poor sleep may reduce self-control and alter sensitivity to rewards through adverse effects on cognitive function.[17] For example, neuroimaging studies have shown that healthy adolescents with poor sleep exhibited altered reward processing and reduced cognitive control compared with adolescents who reported good sleep.[17, 21-23] Likewise, adults exhibited altered reward processing in neuroimaging studies of sleep deprivation.[24-26] Thus, improving sleep may be an important treatment strategy for increasing self-control and reducing risk-taking, such as heavy alcohol consumption, among young adults.

With the exception of our preliminary work, the question of whether improving sleep reduces drinking and alcohol-related risks has only been tested in older adults.[27, 28] In older populations, poor sleep is a well-established alcohol relapse risk factor[29-32] but sleep interventions have yielded mixed results.[29, 33] One challenge for treating sleep problems among older adults with AUDs is that chronic, heavy alcohol exposure can have substantial negative effects on sleep that can persist for two or more years following abstinence.[32] Therefore, it may be more effective to target sleep problems earlier during young adulthood before these negative sleep-alcohol cycles become entrenched. Another potential advantage of sleep interventions is that heavy-drinking young adults are open to information to help them sleep better[34] and sleep interventions address alcohol use as standard care.[35] In our proposed research, we will evaluate whether daily sleep/alcohol self-monitoring + sleep hygiene advice is more effective than sleep hygiene advice alone as well as whether providing personalized feedback based on diaries and sleep/alcohol trackers can lead to further improvements in alcohol outcomes and the mechanisms by which this may occur. The ultimate goal of this research is to examine improved sleep as an effective treatment target for reducing AUD risk in young adults. We will randomly assign participants to 1 of 3 web-based sleep/alcohol self-monitoring + sleep/alcohol data feedback intervention conditions: (1) web-based sleep hygiene advice; (2) web-based sleep hygiene advice + sleep/alcohol diary self-monitoring; or (3) web-based sleep hygiene advice + sleep/alcohol diary self-monitoring + sleep/alcohol data feedback for two weeks of treatment and monitoring

followed by 3 follow up visits. We will measure changes in alcohol consumption and sleep quality in order to address the following aims:

Primary Aim: Examine the effect of sleep condition over time on total drinks consumed over Weeks 4-12, controlling for baseline total drinks. The primary alcohol outcome will be change over time in total drinks consumed. Secondary alcohol outcomes will include changes in total drinks per day, drinks per drinking day, alcohol-related consequences, and estimated blood alcohol levels from the alcohol tracker. We hypothesize that participants in the A+SM+F condition will demonstrate the greatest reductions in total drinks of all 3 conditions which will correspond to significant group by time effects with significant between-group differences in change from baseline to end-point.

Secondary Aim 1: Examine the effect of sleep condition on sleep quality ratings over time, controlling for baseline ratings. The primary sleep outcome will be change in sleep quality ratings (i.e., the perception of how well one has slept) over time. Secondary sleep outcomes will include changes in ratings of sleep-related impairment and sleep tracker quantitative characteristics (i.e., duration, efficiency, % awake, bed/wake times). We hypothesize that participants in the A+SM+F condition will demonstrate the greatest improvements in sleep quality of all 3 conditions which will correspond to significant group by time effects with significant between-group differences in change from baseline to end-point.

Secondary Aim 2: Use descriptive statistics to summarize participants' acceptability ratings of web-based sleep hygiene advice, sleep/alcohol diary self-monitoring, sleep/alcohol tracker use, and personalized sleep/alcohol data feedback. We anticipate that the A+SM+F condition will yield the highest acceptability ratings of all 3 conditions. A review of participants' reactions to personalized sleep feedback will provide insight into what types of feedback and tailored health tips are feasible and useful for heavy-drinking young adults.

Exploratory Aim: Evaluate improvements in sleep quality and self-control (i.e., Go/No Go Task performance) over time as mechanisms of sleep condition effects on total drinks at Month 3. We will calculate individual slope change estimates for sleep quality and self-control and then evaluate these slope estimates as potential mechanisms using a SAS macro outlined by Valeri and VanderWeele.[(69)] This method allows for independent variable X mediator interactions and is suitable for count outcomes (i.e., total drinks). Given the smaller sample size for this exploratory research, we will then evaluate correlations between sleep quality and self-control slope estimates rather than use structural equation modeling to model potential complex pathways among sleep condition, sleep quality, self-control, and drinking.

2. **Probable Duration of Project:** State the expected duration of the project, including all follow-up and data analysis activities.

   It is expected that the study will take 3 years to conduct and analyze.

3. **Background:** Describe the background information that led to the plan for this project. Provide references to support the expectation of obtaining useful scientific data.

Alcohol use disorder (AUD) onset peaks during young adulthood (i.e., 18-25).[1] Compared to older adults, young adults report more frequent and heavier alcohol consumption that is linked to substantial negative consequences including risk of accidental injury, the primary cause of death among young adults.[2-4] Current alcohol intervention strategies for young adults have modest effects[5-7] and young adults rarely self-identify for specialized alcohol treatment.[8, 9] Thus, more work is needed to identify effective alcohol interventions and novel treatment engagement strategies to reduce this substantial public health burden.

One novel approach is to target poor sleep, a common complaint among young adults who drink heavily[10, 11] and an AUD risk factor in young adults.[12-14] Sleep problems in young adults may be due to important developmental changes in sleep that begin with puberty and continue into young adulthood. During this developmental period, there is a need for more sleep[15] and a preference for later bed and wake times,[16] which often conflict with school/work demands and social/cultural obligations.[17] To cope with these conflicts, adolescents and young adults may maintain shorter, more variable sleep schedules putting them at-risk for sleep problems, excessive daytime sleepiness, and other negative consequences.[17-19]

To test poor sleep as a novel treatment target, we conducted the first preliminary test of a sleep intervention in 42 heavy-drinking young adults with sleep concerns (see Preliminary Studies). The study generated a high number of inquiries. Enrolled participants were randomly assigned to 1 of 2 web-based conditions: (1) a sleep intervention that included a brief alcohol intervention or (2) a healthy behaviors intervention with minimal sleep and alcohol advice. All participants completed daily web-based sleep diaries (including tracking of drinks before bedtime) and wore a mobile sleep/wake activity tracker daily to measure outcomes but were not provided any feedback on sleep and alcohol data. Consistent with hypotheses, greater sleep improvement predicted less drinking. However, contrary to expectations, both conditions yielded medium to large improvements in alcohol use, consequences, and ratings of sleep quality and sleep-related impairment. The effects on alcohol outcomes were larger than the small effects observed in typical brief alcohol intervention studies for young adults.[5-7]

These promising results provide preliminary support for utilizing sleep interventions as a novel alcohol treatment strategy for heavy-drinking young adults. Further, the results generated new hypotheses and directions for further refinement of the mobile sleep intervention. The unexpected finding of comparable improvements in sleep and alcohol use across both conditions suggested that common elements, such as brief sleep hygiene advice that includes standard advice to moderate drinking, and daily sleep self-monitoring, including tracking of drinks before bedtime, may have contributed to the outcomes. Sleep hygiene education is effective for improving sleep in young adults.[36, 37] Likewise, self-monitoring can improve a number of health behaviors such as poor sleep and alcohol use.[38-42] Self-monitoring may help individuals learn more about their behavior, identify discrepancies between their goals/standards and actual behavior, and acquire a greater sense of control over their behavior.[43, 44] According to the Theory of Planned Behavior, perceived behavioral control is one factor that can increase intentions to change behavior.[45] To clarify whether sleep monitoring, including monitoring of drinking, is an effective intervention component, a follow-up study is needed with a control condition that does not include self-monitoring

Our qualitative research also yielded insights into ways to improve our intervention. Specifically, participants expressed a desire for personalized feedback about their individual sleep diary/tracker data and their connections with alcohol use in conjunction with health advice tailored to this data. Our prior study used wearable sleep trackers but with the advent of new alcohol biosensors, we can now also use these to provide personalized feedback about sleep/alcohol interactions along with sleep/alcohol diary data. Health feedback is another effective behavior change strategy in line with the Theory of Planned Behavior.(45) Feedback may facilitate behavior awareness and goal setting; ongoing feedback may reinforce behavior change, increase motivation, and enhance self-efficacy.(44) Greater positive beliefs about the outcome of behavior change and greater confidence in one's ability to perform this behavior increase behavior change intentions.(45)

The proposed study is directly responsive to 2 NIAAA strategic directions seeking to identify novel behavioral and integrative treatments for alcohol use disorders and to improve alcohol intervention efforts in young people. We will address the specific calls for studies that incorporate technology and sleep as a treatment target. As a first step, we plan to examine effects of sleep/alcohol self-monitoring and personalized sleep/alcohol data feedback in heavy-drinking young adults. Young adults are the critical population for these studies because sleep patterns and alcohol use are not yet well established and could be readily malleable. Young adults are also the largest consumers of new health technology.(46) A mobile sleep intervention in this cohort could have a substantial public health impact due to the high prevalence of AUDs(1) and infrequent treatment seeking in this population.(8, 9) The information obtained through this research will be used to support 2 future grant applications. The first is a STTR application with a technology partner to make sleep hygiene advice, sleep/alcohol self-monitoring, and sleep/alcohol data feedback completely mobile. Another will be a Phase II test of our mobile sleep intervention combined with psychotherapy and/or pharmacotherapy for more severe young adult drinkers. The ultimate goal of this research is to examine improved sleep as an effective treatment target for reducing AUD risk in young adults.

Preliminary Studies:

<u>Theme 1. Sleep interventions are acceptable and feasible in heavy-drinking young adults and show preliminary efficacy for reducing drinking and alcohol-related consequences.</u>

Dr. Fucito and the research team conducted a preliminary study to investigate a mobile sleep intervention for reducing drinking among heavy-drinking college students (N=42). Participants were randomly assigned to 1 of 2 web-based interventions: (1) "Call it a Night®" and (2) "Healthy Behaviors". <u>"Call it a Night®" (CIAN</u>) incorporated evidence-based content to improve sleep tailored to young adults (i.e., relaxation training, cognitive strategies to target sleep-disruptive beliefs, stimulus control instructions, good sleep hygiene advice) and to reduce drinking (i.e., normative and BAL feedback, moderate drinking guidelines, controlled drinking strategies, effects of alcohol on the body, advice to moderate drinking for improved sleep). "<u>Healthy Behaviors</u>" (HB) had a general health focus with minimal tailoring of content for young adults; basic advice about nutrition, exercise, sleep (i.e., good sleep hygiene advice only) and drinking (i.e., moderate drinking guidelines, advice to moderate drinking for improved sleep, and effects of alcohol on the body). All participants monitored their sleep using daily web-based

diaries (including tracking of drinks before bedtime) and wore a sleep tracker. The program generated ~250 inquiries from college students in 3 months of recruitment. Of the 49 volunteers who met pre-screening eligibility, 86% (n=42) were eligible and enrolled. Treatment completion rates were high (91%). Among all participants, greater improvement in sleep-related impairment tended to predict less drinking at follow-up (b=-.04, SE=.02; Wald χ^2=3.27, p=.07). Contrary to expectations, both interventions significantly reduced total drinks in a typical drinking week and alcohol-related consequences and improved ratings of sleep quality and sleep-related impairment (see Table 2 below). The effects on drinking were larger than small effects observed in typical computerized alcohol interventions for young adults.[6] The HB condition, however, had larger overall effects and yielded greater reductions in total drinks in a heaviest drinking week than the CIAN condition. These results provide promising preliminary support for sleep concerns as a novel treatment target for heavy-drinking young adults. Further, the results suggest that the HB web-based sleep intervention content (i.e., good sleep hygiene advice that includes advice to moderate drinking for better sleep) may be more effective than the CIAN web-based intervention that included the same sleep hygiene advice embedded in additional content related to stress management and stimulus control advice for better sleep.

Theme 2. Heavy-drinking young adults find sleep interventions appealing, are interested in personalized information about sleep and alcohol interactions, and prefer personalized sleep feedback along with tailored advice.

Dr. Fucito and the research team conducted a qualitative study with heavy-drinking college students (N=24) to assess their perceptions of sleep and sleep-alcohol interactions and their intervention preferences.[34] The following common themes emerged from these interviews: strong interest in sleep treatment and interventions that focused on the interaction between sleep and alcohol use, less enthusiasm for alcohol-related intervention content alone unless it had a sufficient rationale for sleep, and strong preference for intervention strategies that included personalized, tailored health feedback and health advice tailored to this data. These findings support a follow-up study to develop and test a personalized sleep/alcohol data feedback plus tailored advice intervention in heavy-drinking young adults.

Table 1. Estimated means (SE) of alcohol consumption, alcohol-related consequences, and subjective sleep-related characteristics by treatment condition and time

	CIAN (n=21)				HB (n=21)			
M (SE)	Intake	Wk 5	3Mo F/U	Effect Size	Intake	Wk 5	3Mo F/U	Effect Size
Drinks Typical Week	17.95 (2.07)	16.34 (2.09)	11.15 (2.15)	d=.58	18.16 (2.51)	13.63 (1.44)	8.56 (1.72)	d=.65
Drinks Heaviest Week	27.50 (4.41)	25.24 (4.97)	20.40 (4.14)	d=.14	24.96 (2.80)	18.01 (1.91)	11.10 (2.17)	d=.47
Alcohol Consequences	15.83 (1.05)	11.02 (1.16)	8.75 (1.07)	d=1.28	17.05 (.83)	9.48 (.59)	7.72 (.52)	d=2.20
Poor Sleep Quality	11.93 (.46)	10.85 (.44)	8.59 (.58)	d=1.29	11.65 (.46)	10.13 (.43)	7.85 (.42)	d=1.66
Sleep-related Impairment	60.52 (1.67)	56.45 (1.31)	51.29 (1.83)	d=1.11	65.41 (1.40)	55.02 (1.89)	50.14 (1.92)	d=2.18

Theme 3. Daily sleep self-monitoring and sleep tracker use are feasible among heavy-drinking young adults. Mobile technology to monitor blood alcohol levels is acceptable to young adults.

In our preliminary study of a mobile sleep intervention for heavy-drinking young adults (N=42), all participants demonstrated adequate adherence to sleep-monitoring activities. Adherence was defined as wearing the sleep tracker 75% of the time and completing 75% of the daily sleep diaries (i.e., 5 days). Adequate adherence during baseline sleep monitoring was a requirement for randomization. Only 1 out of 43 individuals was excluded prior to randomization for not adhering during this baseline period. Of those randomized (N=42), all demonstrated adequate adherence. In our qualitative study to inform initial intervention development, young adults expressed interest in emerging mobile technologies that provide an objective assessment of alcohol consumption.[(34)]

4. **Research Plan:** Summarize the study design and research procedures using non-technical language that can be readily understood by someone outside the discipline. **Be sure to distinguish between standard of care vs. research procedures when applicable, and include any flowcharts of visits specifying their individual times and lengths**. Describe the setting in which the research will take place.

   We will be conducting a study that will examine improved sleep as an effective treatment target for reducing AUD risk in young adults.

   Screening, Intake, and Randomization (Week 0): Participants who meet internet screening eligibility criteria will attend an in-person intake at the SATU clinic of the Connecticut Mental Health Center (CMHC) located at 1 Long Wharf Drive in New Haven to learn about the study, provide informed, voluntary consent, be further evaluated for eligibility, and complete baseline assessments. If found eligible, participants will be randomly assigned to 1 of 3 treatment conditions: Treatment Condition 1: Participants receive only web-based sleep hygiene advice (A); Treatment Condition 2: Participants receive web-based sleep hygiene advice + sleep/alcohol diary self-monitoring (A+SM); Treatment Condition 3: Participants receive web-based sleep hygiene advice + sleep/alcohol diary self-monitoring + sleep/alcohol data feedback (A+SM+F).

Table 2. Sleep Conditions

Condition	Sleep & Alcohol Trackers	Web-based Sleep Hygiene Advice	Sleep/Alcohol Diary Self-Monitoring	Sleep/Alcohol Data Feedback
Web-based Sleep Hygiene Advice (A)	Wear sleep tracker daily on non-dominant wrist<br>Wear alcohol tracker daily on non-dominant wrist	Good sleep habits<br>Advice to moderate drinking to improve sleep; sleep-disruptive effects of alcohol<br>Moderate drinking guidelines<br>Take-home health tips handout	None	None

Web-based Sleep Hygiene Advice +<br>Sleep/Alcohol Diary Self-Monitoring ***(A+SM)***	□Wear sleep tracker daily on non-dominant wrist<br>□Wear alcohol tracker daily on non-dominant wrist	□Good sleep habits<br>□Advice to moderate drinking to improve sleep; sleep disruptive effects of alcohol<br>□Moderate drinking guidelines<br>□Take-home health tips handout	□Daily web-based sleep diaries including alcohol use before bedtime	None
Web-based Sleep Hygiene Advice +<br>Sleep/Alcohol Diary Self-Monitoring + Sleep/Alcohol Data Feedback ***(A+SM+F)***	□Wear sleep tracker daily on non-dominant wrist<br>□Wear alcohol tracker daily on non-dominant wrist□	□Good sleep habits<br>□Advice to moderate drinking to improve sleep; sleep-disruptive effects of alcohol<br>□Moderate drinking guidelines<br>□Take-home health tips handout	□Daily web-based sleep diaries including alcohol use before bedtime	□ Feedback on sleep/alcohol diary and sleep and alcohol tracker data<br>□Feedback on sleep and alcohol data links<br>□Take-home health tips handout tailored to sleep and alcohol data

A randomization ratio of 2:1:1 will be used to assign participants to conditions. Specifically, we will enroll twice as many participants in A+SM+F condition compared to the other 2 conditions. The larger sample will allow us to better evaluate the types of sleep/alcohol data feedback that young adults find helpful and acceptable. There are two types of sleep data: (1) quantitative sleep characteristics from the sleep tracker and sleep diary (e.g., sleep duration, sleep efficiency, percent awake, bed/wake times) and (2) self-report ratings of sleep quality and sleepiness/sleep-related impairment upon waking. In addition, there are two types of alcohol data: (1) estimated blood alcohol level from the alcohol tracker and (2) self-reported alcohol consumption from the sleep diaries. The research assistant will demonstrate how to wear the Fitbit actigraph, the Milo Sensor blood alcohol tracker, how to access the sleep and alcohol diaries, and arrange to pick up the Fitbits, Milo Sensor blood-alcohol trackers, and chargers at the end of each 7-day monitoring period. To encourage compliance, we will compensate participants for each day that they complete daily sleep dairies.

<u>Treatment Phase (Weeks 1-2):</u> For 2 weeks following randomization, participants in all 3 conditions will wear a Fitbit Flex2 sleep tracker and a Milo Sensor blood-alcohol tracker daily. Those assigned to the 2 conditions that involve sleep/alcohol diary self-monitoring (i.e., A+SM; A+SM+F) will complete daily web-based sleep diaries during the 2 weeks following intake.

Table 3. Weekly Sleep and Alcohol Tracker and Sleep/Alcohol Diary Self-Monitoring Activities and Variables (Days 1-7)

Activity	Variables	Instructions	Frequency	Timing
Fitbit Flex2 Sleep Tracker	□Activity/rest patterns (sleep onset/offset, total sleep time, sleep efficiency, % awake)	□Wear Fitbit Flex2 on non-dominant wrist to monitor activity/rest	Days 1-7	Continuously (waterproof device)
Milo Sensor Alcohol Tracker	□Estimated blood alcohol level (BAL)	□Wear Milo Sensor alcohol tracker on non-dominant wrist to monitor BAL	Days 1-7	Continuously (except bathing/ swimming)

Web-based Sleep/Alcohol Diaries	Nocturnal sleep characteristics (sleep onset latency, bed/wake times)<br>Daytime and evening behaviors that affect sleep – *drinks consumed before bedtime*<br>Sleep quality and sleepiness upon waking	Complete diary for sleep and related behaviors the preceding day	Days 1-7	Upon waking

At the end of the first seven-day monitoring period, the research assistant will pick up the Fitbit Flex2 sleep tracker and a Milo Sensor blood-alcohol tracker from the participant. Upon receipt of the sleep trackers, we will download participants' scored sleep and alcohol use data from the prior week to include in the web-based sleep hygiene program and tailor the take-home health tips handouts that participants will receive the next day at each in-person visit. The next day the participant will return for an in-person visit and will receive empirically-supported brief sleep hygiene advice via the web-based program from our prior study. At the Week 1 visit, participants will view module 1 (~10 minutes) which covers good sleep hygiene advice focused on reducing sleep-disruptive behaviors (e.g., caffeine, nicotine use; poor diet) including alcohol use. Module 1 will include advice to moderate drinking for better sleep, moderate drinking guidelines for men and women, the sleep-disruptive effects of alcohol, and drinking reduction strategies. Participants will receive a take-home handout that summarizes the health tips provided in the module at the end of the visit. If the participant is in the treatment condition that involves personalized sleep/alcohol data feedback (A+SM+F), they will receive this information along with the web-based sleep hygiene advice program. In the A+SM+F condition, feedback will be derived prior to the visits from all possible available data (i.e., sleep diary data, including drinks before bedtime, and sleep and alcohol tracker data) and will be embedded in the web-based sleep hygiene modules. In addition, health tips tailored to this sleep/alcohol data will be added to their take-home handouts.

This process will be repeated for another seven-day period, leading to the next in-person visit at the end of week 2 where the participant will receive sleep hygiene advice through viewing module 2 (~10 minutes), which will cover good sleep hygiene advice focused on establishing good sleep habits (i.e., ideal light, temperature, and sound settings for good sleep, bedtime routines, and limiting other activities in the bed/bedroom). Participants will again receive a take-home handout that summarizes the health tips provided in the module and will complete treatment evaluation exit interviews with Dr. Fucito or Dr. DeMartini. These interviews will be audiotaped with participants' consent.

Follow-Up Phase: (Weeks 4, 8, 12): Participants will attend a 1 follow-up visit at each of these time points for a total of 3 follow-up visits: Weeks 4, 8, and 12 after starting treatment. At these visits, participants will complete a number of questionnaires that will ask about and assess sleep, mood, health behaviors, alcohol use, psychological characteristics, and will complete a computer task (See Table 3 Variables/Measures below). Participants will also be asked about their use of the tailored health tips based on their sleep data and whether any of the sleep tips were helpful.

Figure 1: Study Flow Chart

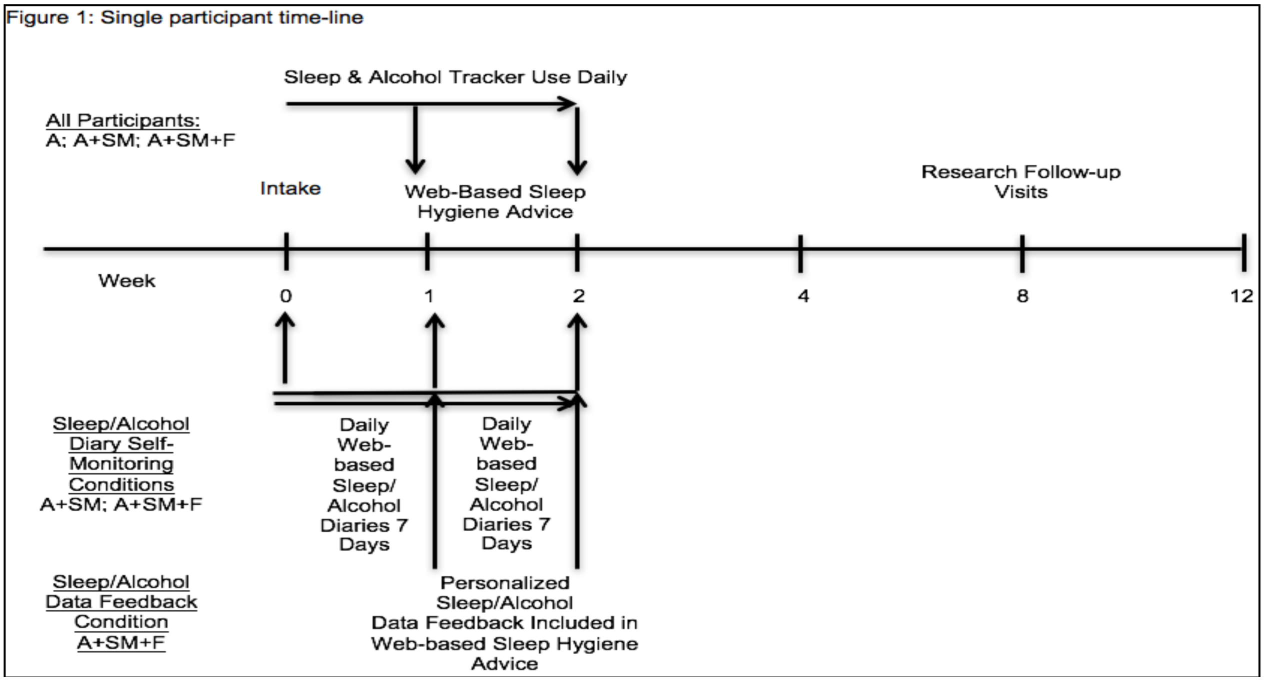

Table 4: Variables/Measures

Variables	Assessments	Intake	Diary Self-Monitoring	Alcohol/ Sleep Trackers	Weeks 1, 2, 4, 8, 12
**Eligibility/ Lab/ Medical**	Demographics	X			
	DSM-V Diagnoses	X			
	Urine Drug Screen/Breath Alcohol	X			X
	AUDIT	X			
	Endorse Sleep Concerns	X			
**Alcohol Outcomes**	Timeline Followback	X			X
	Alcohol-Related Consequences	X			4, 8, 12
	Tracker Blood Alcohol Level			X	
**Sleep Outcomes**	PROMIS™ Sleep-related Impairment	X			4, 8, 12
	PROMIS™ Sleep Disturbance	X			4, 8, 12
	Pittsburgh Sleep Diary		X		
	Profile of Mood States	X			4, 8, 12
	Munich Chronotype and Horne-Ostberg Morningness/Eveningness	X			
	FitBit: sleep onset/offset; total sleep time; sleep efficiency; % awake			X	
**Self-Control**	Go/No Go Task	X			4, 8, 12
**Feasibility**	Adherence (diaries, trackers, tips)		X	X	X
**Acceptability**	Treatment evaluation; exit interview				2

Assessments:

Eligibility, Laboratory, Medical Assessments:

Socio-demographic information will be assessed with interviews and self-report forms that provide data on age, race, socioeconomic and marital status, and educational and occupational levels.

Diagnostic and Substance Use History will be assessed by Drs. Fucito and DeMartini, licensed clinical psychologists, using the Structured Clinical Interview for DSM-V[(61)] (i.e., current and past substance use disorders, other current Axis I psychiatric diagnoses). An **Alcohol Use Disorders Identification Test**, a reliable, valid alcohol use screener, will be administered to confirm the inclusion criterion of at-risk drinking based on recommended AUDIT-C cut-off scores for young adults.[(48)]

Urine Drug Toxicology will be based on a JANT Pharmaceuticals urine test kit for opiates, cocaine, barbiturates, amphetamines, benzodiazepines, or phenylcyclidine. Positive tests are an exclusion criterion.

Breath Alcohol Concentrations for in-person visits will be determined using a hand-held breathalyzer unit - an Alcohol-Sensor III (Intoximeter Inc., St. Louis, MO). Participants need to test negative to provide consent at intake and need to test <.04% at subsequent in-person treatment and assessment visits.

Drinking Measures

Timeline Follow-back Interview (TLFB) is a standardized, validated, and reliable experimenter-administered interview that will be used to obtain quantity and frequency estimates of alcohol consumption for a 90-day period prior to enrollment in the study and monthly following intake for a total of 3 months.[(51)] Calendar prompts and memory aids (e.g., holidays) are used to facilitate accurate recall of substance use during the targeted period. We will derive the following 30-day summary variables from the TLFB for intake and Weeks 4, 8, and 12: total drinks, drinks per day, drinks per drinking day.

Alcohol-Related Problems will be assessed at intake, Weeks 4, 8, and 12 using the Young Adult Alcohol Consequences Questionnaire (YAACQ), a reliable, valid measure of 48 consequences that may have occurred due to alcohol consumption that are predictive of drinking persistence among young adults.[(62)]

Blood Alcohol Concentrations during at-home assessment will be determined using the Milo Sensor alcohol tracker, a wrist-worn alcohol biosensor, which provides a continuous measure of participants' blood alcohol levels. The device works with a smartphone to track this data. We will turn off access to the data so that participants do not receive immediate feedback about their blood alcohol levels. We will also set up the application so that it is not directly linked to participants but rather to their participant numbers. Data is downloaded to a secure, password-protected server administered by Milo Sensors. Any data stored on the device will be immediately deleted after download. We will download the data using our research computers after collecting the devices from participants.
Participants will wear the alcohol tracker on their non-dominant hand from 8pm to 8am daily. We will derive the following variables from the tracker: peak blood alcohol level, average blood

alcohol level.

Sleep and Sleep-Related Characteristics:

Sleep Concerns. Participants will be asked at screening if they are concerned about their sleep using a dichotomous item (i.e., yes/no) developed for our initial study. Positive endorsement is an inclusion criterion.

NIH PROMIS™ Sleep-Related Impairment is a validated, reliable measure of perceived alertness, sleepiness, and tiredness during waking hours and functional impairments due to sleep problems.[(63)] Participants' scores can be compared to national norms. The PROMIS-SRI will be assessed at intake and Weeks 4, 8, and 12.

NIH PROMIS™ Sleep Disturbance is a validated, reliable measure of perceived sleep quality/satisfaction and difficulty initiating/maintaining sleep.[(63)] Participants' scores can be compared to national norms. The PROMIS-SD will be administered at intake and Weeks 4, 8, and 12.

Profile of Mood States-Short Form is a validated, reliable 37-item measure of positive and negative mood that yields 6 subscales and a total score.[(64)] The POMS-SF will be assessed at intake and Weeks 4, 8, and 12. Sleep improvement may cause mood changes that could affect alcohol outcomes.

Munich Chronotype/Horne-Ostberg Morningness-Eveningness Questionnaires are reliable, valid assessments of participants' chronotype and morning/evening preference that will be administered at intake.[(65)]

Pittsburgh Sleep Diary is a well-validated assessment of daytime sleep-related behaviors and nocturnal sleep characteristics.[(66)] Participants assigned to conditions that include sleep self-monitoring will complete daily web-based Pittsburgh Sleep Diaries in the mornings to track daytime sleep-related behaviors and nocturnal sleep characteristics of the preceding day. Diaries will include questions about caffeine and alcohol use as well as ratings of sleep quality and sleepiness upon waking.

Actigraphy is a valid, reliable methodology used in research to objectively estimate sleep/wake activity. All participants will wear the Fitbit Flex2, a commercially available wrist-worn device that incorporates actigraphy technology. The Fitbit Flex2 is worn on the non-dominant wrist to obtain objective measures of sleep/wake activity. Validation studies provide evidence of its reliability and validity relative to well-validated ambulatory (i.e., actigraphy) and laboratory methods (i.e., polysomnography) for assessing sleep. [(52-54)]. The following variables will be derived: sleep onset/offset (i.e., bed/wake time), total sleep time (i.e., sleep duration), sleep efficiency, and percent awake. The device works with a smartphone to track this data. We will turn off access to the data so that participants do not receive immediate feedback about their sleep and activity levels. We will also set up the application so that it is not directly linked to participants but rather to their participant numbers. Data is downloaded to a secure, password-protected server administered by Fitbit. Any data stored on the device will be immediately

deleted after download. We will download the data using our research computers after collecting the devices from participants.

Self-Control:

Go/No Go Task, a reliable, valid computerized task that will be used to assess self-control, specifically the ability to inhibit an inappropriate response. The ability to inhibit responding has been shown to be related to alcohol use and to be sensitive to changes in sleep.[67, 68] In the Go/No Go Task, participants are instructed to respond when certain stimuli are present (i.e., typically a letter or number) and then to refrain from responding when the target stimuli are not present. The task will be administered at intake and Weeks 4, 8, and 12.

Intervention Component Feasibility and Acceptability:

We will evaluate participant use metrics to determine intervention component feasibility (i.e., diary and tracker adherence, use of health tips during treatment and follow-up). At Week 2, all participants will complete an end of treatment evaluation form. We will also interview participants in A+SM+F to evaluate their reactions to and preferences for sleep/alcohol data monitoring and feedback.

5. Genetic Testing N/A ☒

A. Describe

i. the types of future research to be conducted using the materials, specifying if immortalization of cell lines, whole exome or genome sequencing, genome wide association studies, or animal studies are planned

ii. the plan for the collection of material or the conditions under which material will be received

iii. the types of information about the donor/individual contributors that will be entered into a database

iv. the methods to uphold confidentiality

B. What are the conditions or procedures for sharing of materials and/or distributing for future research projects?

C. Is widespread sharing of materials planned?

D. When and under what conditions will materials be stripped of all identifiers?

E. Can donor-subjects withdraw their materials at any time, and/or withdraw the identifiers that connect them to their materials?

i. How will requests to withdraw materials be handled (e.g., material no longer identified: that is, anonymized) or material destroyed)?

F. Describe the provisions for protection of participant privacy

G. Describe the methods for the security of storage and sharing of materials

6. **Subject Population:** Provide a detailed description of the types of human subjects who will be recruited into this study.

Participants will be up to 120 male and female volunteers (plus up to 5 pilot subjects), 18-25 years of age, who report ≥4 heavy drinking occasions per month in the past 90 days and report having concerns about their sleep.

Participants must meet inclusion/exclusion criteria as listed below. Based on the demographics of New Haven and the surrounding communities obtained from census data, we anticipate the following breakdown: White (not Hispanic) 70%, Black 17%, White (Hispanic) 10%, Asian/Asian Indian 3%.

7. **<u>Subject classification:</u>** Check off all classifications of subjects that will be <u>specifically recruited for enrollment</u> in the research project. Will subjects who may require additional safeguards or other considerations be enrolled in the study? If so, identify the population of subjects requiring special safeguards and provide a justification for their involvement.

☐Children ☒ Healthy ☐Fetal material, placenta, or dead fetus
☐Non-English Speaking ☐ Prisoners ☐Economically disadvantaged persons
☐Decisionally Impaired ☐ Employees ☐Pregnant women and/or fetuses
☒Yale Students ☐ Females of childbearing potential

NOTE: Is this research proposal designed to enroll children who are wards of the state as potential subjects?
Yes ☐ No ☒

8. **<u>Inclusion/Exclusion Criteria</u>:** What are the criteria used to determine subject inclusion or exclusion?

<u>Participants will be eligible if they:</u>

(1) 18-25 years of age;
(2) report ≥ 4 drinking occasions per month in the past 90 days (i.e., ≥5 drinks on 1 occasion for men; ≥4 for women);
(3) report having concerns about their sleep;
(4) willing/able to complete daily sleep diaries and wear sleep and alcohol trackers;
(5) report Alcohol Use Disorders Identification Test – Consumption (AUDIT-C) scores indicative of risk of harm from drinking (i.e., ≥7 and ≥5 for men and women, respectively);[48]
(6) read and understand English;
(7) have a smartphone that can be used to sync tracker data. An estimated 86% of young adults own a smartphone.[49]

Participants will be excluded from study if they:

(1) history of a sleep disorder;
(2) night or rotating shift work; travel beyond 2 time zones in month prior and/or planned travel

beyond 2 time zones during study participation;
(3) meet criteria for an alcohol use disorder in the past 12 months that is clinically severe defined by: *a) a history of seizures, delirium, or hallucinations during alcohol withdrawal; b) report drinking to avoid withdrawal symptoms or have had prior treatment of alcohol withdrawal; c) have required medical treatment of alcohol withdrawal in the past 6 months;*
(4) currently enrolled in alcohol or sleep treatment;
(5) exhibit current psychiatric illness (i.e., bipolar disorder, schizophrenia, major depression, panic disorder, borderline personality disorder, organic mood or mental disorders, or suicide or violence risk) by history or psychological examination;
(6) current DSM-V substance use disorder or a positive urine drug screen for opiates, cocaine, barbiturates, benzodiazepines, amphetamines, or phenylcyclidine. *A positive urine drug screen for marijuana use, among non-dependent marijuana users will not be an exclusion criterion. non-dependent marijuana use is very common among heavy-drinking young adults;*[(50)] *exclusion would limit recruitment and external validity. We will collect marijuana/caffeine use that can be considered as moderators or mediators in secondary analyses.*

9. How will **eligibility** be determined, and by whom?

   The research staff will evaluate initial eligibility; the research coordinator will evaluate eligibility for the study and screen based on inclusion and exclusion criteria. The research staff will evaluate initial eligibility; the research coordinator will evaluate eligibility for the study and screen based on inclusion and exclusion criteria. Dr. Fucito or Dr. DeMartini will conduct a structured clinical interview to assess for psychiatric disorders including alcohol and substance use disorders.

10. **Risks:** Describe the reasonably foreseeable risks, including risks to subject privacy, discomforts, or inconveniences associated with subjects participating in the research.

    <u>Sleep Intervention Components:</u>
    Sleep and alcohol tracker use poses minimal risk. The sleep and alcohol information contained in the web-based sleep hygiene advice modules poses minimal risk. A great deal of this information is also available to young adults through external health websites. Our experience providing brief alcohol and sleep interventions to young adults and cognitive-behavioral sleep interventions indicates that they are well tolerated. Our research group has several PhD-level clinical psychologists available for consultation should the need arise. Participants will be monitored regularly during in-person research visits.

    <u>Interviews and Self-Reports:</u>
    Research interviews and assessments, including completing sleep diaries, are all non-invasive and should add no risk. The major disadvantage is the time it takes to complete them and possible breach of confidentiality. Our research group's experience with these measures indicates that they are acceptable to patients. Drs. Fucito and DeMartini, licensed clinical psychologists, will be available to meet with individuals and will provide staff with consultation should the need arise. Careful efforts aimed at maintaining confidentiality will be made, which are described below, and only participants' study numbers will be recorded on the forms themselves in order to protect confidentiality.

Urine Collection:
Screening urine collections are performed primarily as safeguards to participants and should add no risks other than those normally associated with these procedures. Urine test kits used to screen for illicit drugs will not be retained for further analysis.

Audiotaping of Exit Interviews:
Audiotaping of interviews is necessary to evaluate participants' reactions to personalized feedback and tailored health advice.

11. **Minimizing Risks:** Describe the manner in which the above-mentioned risks will be minimized.

Audiotaping of Exit Interviews: To assure the confidentiality and protection of participants with respect to these procedures, the following steps will be taken:

- Participants have the right to refuse taping. Participants who consent to taping will be informed that they have the right to stop taping at any time.
- All taping will take place in a designated interview room in our research suite. Only Drs. Fucito and DeMartini will tape interviews.
- Sessions will be taped using digital recorders and saved on a password protected secure server. Digital file names will only be identified by participants' study numbers and session number.
- Sessions will then be logged and uploaded to a secure password protected server in a secure research office at SATU at 1 Long Wharf Drive, New Haven, CT.
- Access to digital recordings will be limited to key study personnel.

Effective screening will exclude all participants who would be at greater risk for complications from sleep interventions and/or study participation because of medical or specific psychiatric illnesses. Drs. Fucito and DeMartini, licensed clinical psychologists, will evaluate all potential participants for inclusion. If participants are not eligible for the study and/or request further assistance with alcohol use or sleep once the study is completed, appropriate referrals will be provided. Upon study enrollment, numerous safeguards will be used to monitor participants. Participants will be seen weekly during treatment and 2/3 will complete daily diaries of their sleep that can be monitored remotely by Study Staff. Plus, all will wear alcohol and sleep trackers. Alcohol tracker data can be monitored remotely. Dr. Fucito, a licensed clinical psychologist with clinical expertise in sleep and alcohol, including interventions for young adults, will be available to meet with participants. Dr. Fucito will also provide participants with treatment referrals if they are interested.

Right to privacy for participation in this research will be protected through alphanumeric coding of data (in place of names) and proper storage of research records, including treatment exit interviews. Collected materials will be maintained via an alphanumeric reference system maintained by Dr. Fucito. Participants' names will appear only on the consent form, the HIPAA authorization form, and a master list maintained in a physically locked file that is separate from research data. Our data collection and management procedures are fully compliant with HIPAA. Access will be limited to personnel intimately involved in the study. A Certificate of Confidentiality will also be obtained from the National Institutes of Health to protect access to the records. However, participants will also be told that if they present with suicidal or homicidal

ideation and/or report any form of child/elder abuse or report plans to damage property then we will have to report this to the appropriate authorities and/or provide them with referrals for immediate treatment. Electronic data will be de-identified and password protected. Only members of the study team will have access to the physical or electronic data. Fitbit and Milo Sensors data are also protected through secure, password-protected servers.

12. **Data and Safety Monitoring Plan:** Include an appropriate Data and Safety Monitoring Plan (DSMP) based on the investigator's risk assessment stated below. (Note: the HIC will make the final determination of the risk to subjects.)
    a. What is the investigator's assessment of the overall risk level for subjects participating in this study? Minimal

b. If children are involved, what is the investigator's assessment of the overall risk level for the children participating in this study?

No children under 18 will be enrolled in this study.

c. Include an appropriate Data and Safety Monitoring Plan. Examples of DSMPs are available here http://your.yale.edu/policies-procedures/forms/420-fr-01-data-and-safety-monitoring-plans-templates for

i. Minimal risk
ii. Greater than minimal

d. For multi-site studies for which the Yale PI serves as the lead investigator:

i. How will adverse events and unanticipated problems involving risks to subjects or others be reported, reviewed and managed? N/A
ii. What provisions are in place for management of interim results? N/A
iii. What will the multi-site process be for protocol modifications? N/A

Dr. Fucito, the principal investigator is responsible for monitoring the data, assuring protocol compliance, and conducting monthly safety reviews. During the review process, Dr. Fucito, will evaluate whether the study should continue unchanged, require modification/amendment, or close to enrollment.

Dr. Fucito, the Institutional Review Board (IRB) or NIH have the authority to stop or suspend the study or require modifications.

This protocol presents minimal risks to the subjects and Unanticipated Problems Involving Risks to Subjects or Others (UPIRSOs), including adverse events, are not anticipated. In the unlikely event that such events occur, Reportable Events (which are events that are serious or life-threatening and unanticipated (or anticipated but occurring with a greater frequency than expected) and possibly, probably, or definitely related) or Unanticipated Problems Involving Risks to Subjects or Others that may require a temporary or permanent interruption of study activities will be reported immediately (if possible), followed by a written report within 48 hours of Dr. Fucito becoming aware of the event to the IRB (using the appropriate forms from the website) and any appropriate funding and regulatory agencies.

Dr. Fucito will apprise fellow investigators and study personnel of all UPIRSOs and adverse events that occur during the conduct of this research project. Dr. Fucito will hold weekly study meetings with investigators to review study progress, including any adverse events. Investigators who are not present at these meetings will be alerted via email by Dr. Fucito.
The protocol's research monitor(s), e.g., the Yale IRB and NIH will be informed of *any adverse events that meet these 3 criteria: (1) unexpected, (2) related/possibly related to study participation, and (3) places subjects or others at greater risk of harm than previously known/recognized. These* adverse events will be reported within 48 hours of the event becoming known to Dr. Fucito.

13. **Statistical Considerations:** Describe the statistical analyses that support the study design.

The goal of the Primary Aim is to examine the effect of sleep condition over time on total drinks consumed over Weeks 4-12, controlling for baseline total drinks. For this analysis, we will evaluate changes in scores using a mixed model repeated measures analysis with sleep condition and sex as between-subject factors and time as a within-subject factor. We will also test changes in secondary alcohol outcomes: drinks per day, drinks per drinking day, alcohol-related consequences, and estimated blood alcohol level, controlling for baseline alcohol outcomes, for which we will adjust for multiple comparisons. A mixed model will account for the correlation in alcohol outcomes measured in the same individual and will allow us to use all available data on individuals. The best-fitting variance-covariance structure will be selected using Schwartz-Bayesian Information Criterion (BIC). Time will be considered as a categorical factor but we will also evaluate whether alcohol outcomes change linearly by sleep condition over time. Where appropriate, we will apply transformations (e.g. log) to the dependent measures to comply with model assumptions or we will employ alternative methods (i.e. resampling, nonparametric tests). <u>Expected Results:</u> We hypothesize that participants in the A+SM+F condition will demonstrate the greatest reductions in total drinks of all 3 conditions which will correspond to significant group by time effects with significant between-group differences in change from baseline to end-point.

Statistical Analyses for Secondary Aim
The goal of the Secondary Aim 1 is to examine the effect of sleep condition on sleep quality ratings over time, controlling for baseline ratings. We will use mixed model repeated measures analysis as described above. We will also test changes in secondary sleep outcomes: ratings of sleep-related impairment and sleep quantitative outcomes (i.e., duration, efficiency, % awake, bed/wake times), controlling for baseline responses. <u>Expected Results:</u> We hypothesize that participants in the A+SM+F condition will demonstrate the greatest improvements in sleep quality of all 3 conditions which will correspond to significant group by time effects with significant between-group differences in change from baseline to end-point.

For Secondary Aim 2, we will use descriptive statistics to summarize participants' acceptability ratings of web-based sleep hygiene advice, sleep/alcohol diary self-monitoring, sleep/alcohol tracker use, and personalized sleep/alcohol data feedback. We anticipate that the A+SM+F condition will yield the highest acceptability ratings of all 3 conditions. A review of participants'

reactions to personalized sleep feedback will provide insight into what types of feedback and tailored health tips are feasible and useful for heavy-drinking young adults.

For the Exploratory Aim, we will evaluate improvements in sleep quality and self-control (i.e., Go/No Go Task performance) over time as mechanisms of sleep condition effects on total drinks at Month 3. We will calculate individual slope change estimates for sleep quality and self-control and then evaluate these slope estimates as potential mechanisms using a SAS macro outlined by Valeri and VanderWeele. (69) This method allows for independent variable X mediator interactions and is suitable for count outcomes (i.e., total drinks). Given the smaller sample size for this exploratory research, we will then evaluate correlations between sleep quality and self-control slope estimates rather than use structural equation modeling to model potential complex pathways among sleep condition, sleep quality, self-control, and drinking.

SECTION II: RESEARCH INVOLVING DRUGS, BIOLOGICS, RADIOTRACERS, PLACEBOS AND DEVICES

If this section (or one of its parts, A or B) is not applicable, check off N/A and delete the rest of the section.

A. RADIOTRACERS **☒N/A**

If NO, an FDA issued IND is required for the investigational use unless RDRC assumes oversight.

2.

B. DRUGS/BIOLOGICS **☒N/A**

2.

B. DEVICES **☒N/A**

SECTION III: RECRUITMENT/CONSENT AND ASSENT PROCEDURES

1. **Targeted Enrollment: Give the number of subjects:**

a. Targeted for enrollment at Yale for this protocol: 120 + 5 pilot subjects

b. If this is a multi-site study, give the total number of subjects targeted across all sites:

2. **Indicate recruitment methods below.** Attach copies of any recruitment materials that will be used.

☒ Flyers	☒ Internet/web postings	☐ Radio
☒ Posters	☐ Mass email solicitation	☐ Telephone
☐ Letter	☐ Departmental/Center website	☐ Television
☐ Medical record review*	☐ Departmental/Center research boards	☐ Newspaper
☐ Departmental/Center newsletters	☐ Web-based clinical trial registries	☒ Clinicaltrails.gov

☐ YCCI Recruitment database ☒ Social Media (Twitter/Facebook):
☐ Other:

*** Requests for medical records should be made through JDAT as described at** http://medicine.yale.edu/ycci/oncore/availableservices/datarequests/datarequests.aspx

3. Recruitment Procedures:

a. Describe how potential subjects will be identified.
b. Describe how potential subjects are contacted.
c. Who is recruiting potential subjects?

We will recruit most participants through online advertising/social media (e.g., Facebook, Google): methods used successfully by Drs. Fucito and O'Malley to recruit young adult drinkers. We will also display notices around the New Haven community and 10 local colleges.

Interested individuals who contact investigators by telephone or by email will be directed to a secure website link to complete a brief, 5 min, pre-screening survey. Web-based advertisements will also direct volunteers to the brief web-based pre-screener. Before completing the pre-screener, volunteers will provide informed consent. At each stage of screening, individuals will have the opportunity to ask questions about the study. Following completion of the web-based pre-screener, research staff will contact potential participants and inform them of their initial eligibility status. Potential participants who meet initial eligibility criteria will attend an in-person intake to meet with the Research Coordinator who will obtain informed consent and screen for inclusion/exclusion criteria.

4. Assessment of Current Health Provider Relationship for HIPAA Consideration:
Does the Investigator or any member of the research team have a direct existing clinical relationship with any potential subject?

☐Yes, all subjects
☐Yes, some of the subjects
☒No

If yes, describe the nature of this relationship.

5. <u>Request for waiver of HIPAA authorization:</u> (When requesting a waiver of HIPAA Authorization for either the entire study, or for recruitment purposes only. Note: if you are collecting PHI as part of a phone or email screen, you must request a HIPAA waiver for recruitment purposes.)

Choose one:

☐ For entire study
☒ For recruitment/screening purposes only
☐ For inclusion of non-English speaking subject if short form is being used and there is no translated HIPAA research authorization form available on the University's HIPAA website at hipaa.yale.edu.

i. Describe why it would be impracticable to obtain the subject's authorization for use/disclosure of this data:

ii. If requesting a waiver of **signed** authorization, describe why it would be impracticable to obtain the subject's signed authorization for use/disclosure of this data:

The majority of potential participants first contact research staff via the web or phone. In our experience, very few individuals present in person to inquire about the study and complete the pre-screening process. Potential participants who first contact research staff through the webscreener, are not permitted to advance to the screening survey until they select the "yes" option agreeing that they have read and understood the disclaimer and consent to participate in the pre-screening process. The disclaimer describes the purpose of the webscreener including information about confidentiality, and provides the option for the individual to exit the webscreener and to conduct the pre-screening by phone.

The collection of PHI is limited to information that is necessary to confirm basic eligibility such as age, information to contact the individual with their eligibility status such as email and phone number (the latter is optional, the former is not), and IP address. Once the participant's eligibility has been reviewed and they have been contacted, their webscreener entry will be deleted.

During the phone screening process, individuals provide verbal consent. The collection of PHI is limited to information that is necessary to confirm basic eligibility such as age. If an individual is not eligible, no other PHI or contact information is obtained. If an individual meets pre-screening eligibility and would like to schedule an in-person screening appointment, his/her name and phone number is then obtained. This information is obtained in order to identify the individual on the day of screening (i.e., potential participants need to provide valid identification at intake) and to provide the individual with a reminder call the day before the appointment. It would not be practical to have potential participants provide signed authorization at the time of recruitment over the phone or via the webscreener. Before completing the pre-screener, volunteers will provide informed consent by clicking yes or no that they understand the material presented and agree to continue and participate in the webscreener.

It presents an extra hurdle and potential waste of time for potential individuals when inquiring about the study. This system provides a more efficient method for pre-screening individuals and ensuring that the majority of individuals who are scheduled for an in-person intake appointment are likely to be eligible.

The investigator assures that the protected health information for which a Waiver of Authorization has been requested will not be reused or disclosed to any person or entity other than those listed in this application, except as required by law, for authorized oversight of this research study, or as specifically approved for use in another study by an IRB.

Researchers are reminded that unauthorized disclosures of PHI to individuals outside of the Yale HIPAA-Covered entity must be accounted for in the "accounting for disclosures log", by subject

name, purpose, date, recipients, and a description of information provided. Logs are to be forwarded to the Deputy HIPAA Privacy Officer.

6. **Process of Consent/Assent:** Describe the setting and conditions under which consent/assent will be obtained, including parental permission or surrogate permission and the steps taken to ensure subjects' independent decision-making.

   Informed consent will be obtained from each participant at entry into the study. The entire consent form will be reviewed in detail with the participant in a private, one-on-one setting at the intake appointment. All risks and potential benefits will be described and discussed. Any questions that the participant may have will be addressed. If the participant wishes, they may take the consent form home and consider it further before signing. They may also request to speak to anyone on the research team about questions they have or to consult others, including their physician and family members. Once the participant has signed the consent, they may withdraw consent at any time. Informed consent must be obtained prior to performance of any protocol specific procedures.

7. **Evaluation of Subject(s) Capacity to Provide Informed Consent/Assent:** Indicate how the personnel obtaining consent will assess the potential subject's ability and capacity to consent to the research being proposed.

   We will not be enrolling participants with limited decision-making capacity. We plan to exclude individuals with current serious psychiatric or medical illnesses. During the consenting process, the research coordinator will read and review the consent form with the prospective participant. The research coordinator will then ask the potential participant various questions about the consent form and study protocol to ensure the prospective participant sufficiently understands the study and the nature of their consent to participate.

8. **Non-English Speaking Subjects:** Explain provisions in place to ensure comprehension for research involving non-English speaking subjects. If enrollment of these subjects is anticipated, translated copies of all consent materials must be submitted for approval prior to use.
   We will not enroll non-English speaking subjects.

   As a limited alternative to the above requirement, will you use the short form* for consenting process if you unexpectedly encounter a non-English speaking individual interested in study participation and the translation of the long form is not possible prior to intended enrollment?
   YES ☐ NO ☒

Note* If more than 2 study participants are enrolled using a short form translated into the same language, then the full consent form should be translated into that language for use the next time a subject speaking that language is to be enrolled.

Several translated short form templates are available on the HRPP website (yale.edu/hrpp) and translated HIPAA Research Authorization Forms are available on the HIPAA website (hipaa.yale.edu). If the translation of the short form is not available on our website, then the

translated short form needs to be submitted to the IRB office for approval via modification prior to enrolling the subject. ***Please review the guidance and presentation on use of the short form available on the HRPP website.***

If using a short form without a translated HIPAA Research Authorization Form, please request a HIPAA waiver in the section above.

9. Consent Waiver: In certain circumstances, the HIC may grant a waiver of signed consent, or a full waiver of consent, depending on the study. If you will request either a waiver of consent, or a waiver of signed consent for this study, complete the appropriate section below.

☐Not Requesting any consent waivers

☐Requesting a waiver of <u>signed</u> consent:
☒ Recruitment/Screening only *(if for recruitment, the questions in the box below will apply to recruitment activities only)*
☐ Entire Study (Note that an information sheet may be required.)

For a waiver of signed consent, address the following:

- Would the signed consent form be the only record linking the subject and the research? YES ☒ NO ☐
- Does a breach of confidentiality constitute the principal risk to subjects? YES ☒ NO ☐

OR

- Does the research pose greater than minimal risk? YES ☐ NO☒
- Does the research include any activities that would require signed consent in a non-research context? YES ☐ NO ☒

☐ Requesting a waiver of consent:
☐ <u>Recruitment/Screening</u> only *(if for recruitment, the questions in the box below will apply to recruitment activities only)*
☐ <u>Entire Study</u>

For a full waiver of consent, please address all of the following:

- Does the research pose greater than minimal risk to subjects?
  ☐ Yes *If you answered yes, stop. A waiver cannot be granted.*

  ☐ No

- Will the waiver adversely affect subjects' rights and welfare? YES ☐ NO☐
- Why would the research be impracticable to conduct without the waiver?
- Where appropriate, how will pertinent information be returned to, or shared with subjects at a later date?

Section IV: Protection of Research Subjects

Confidentiality & Security of Data:

1. What protected health information (medical information along with the HIPAA identifiers) about subjects will be collected and used for the research?

   Identifiable information including participant's name, address, phone number, and date of birth, will be collected and used to enroll and contact participants. It will only be used for this purpose. This information will be stored in locked cabinet apart from the research records.

2. How will the research data be collected, recorded and stored?

   Research data will be collected using interviews, self-reports, wearable devices, and computer tasks. All identifiable information will be stored in a locked research cabinet. All participants will be assigned a study participant number. Subsequently, participants will be identified in the Case Report Forms (CRFs) only by that number and an encoded version of their initials (i.e., John Doe = JDO). A list of numbers and the corresponding names will be maintained by Dr. Fucito and stored in a locked research cabinet.

3. How will the digital data be stored? ☐CD ☐DVD ☐Flash Drive ☐Portable Hard Drive ☒Secured Server ☒Laptop Computer ☒Desktop Computer ☐Other

4. What methods and procedures will be used to safeguard the confidentiality and security of the identifiable study data and the storage media indicated above during and after the subject's participation in the study?

   Several steps will be taken to safeguard the confidentiality of subjects and their data. All research data that is collected will be assigned a study participant number and that number will only identify participants in digital databases. The names of participants will not be associated with this data and assessments will be maintained according to participant study number. A master list connecting participant study numbers to participant names will be kept in a locked file cabinet where it can only be accessed by senior level project staff. Any information published as a result of the study will be such that it will not permit identification of any participant.

Right to privacy for participation in this research will be protected through alphanumeric coding of data (in place of names) and proper storage of research records, including treatment exit interviews. Collected materials will be maintained via an alphanumeric reference system maintained by Dr. Fucito. Participants' names will appear only on the consent form, the HIPAA authorization form, and a master list maintained in a physically locked file that is separate from research data. Our data collection and management procedures are fully compliant with HIPAA. Access will be limited to personnel intimately involved in the study. A Certificate of Confidentiality will also be obtained from the National Institutes of Health to protect access to the records. However, participants will also be told that if they present with suicidal or homicidal ideation and/or report any form of child/elder abuse or report plans to damage property then we will have to report this to the appropriate authorities and/or provide them with referrals for immediate treatment. Electronic data will be de-identified and password protected. Only members of the study team will have access to the physical or electronic data.

For the wearable devices, data will only be linked to participants' study numbers not the participants themselves. Participants will not be able to access their Fitbit sleep data or Milo Sensor alcohol use data. The data will be downloaded to secure, password-protected servers. After downloading the data, the data will be immediately deleted from the devices.

All investigators and key personnel have taken the required Yale University HIPAA training.

All portable devices must contain encryption software, per University Policy 5100. If there is a technical reason a device cannot be encrypted please submit an exception request to the Information Security, Policy and Compliance Office by clicking on url http://its.yale.edu/egrc or email it.compliance@yale.edu

5. What will be done with the data when the research is completed? Are there plans to destroy the identifiable data? If yes, describe how, by whom and when identifiers will be destroyed. If no, describe how the data and/or identifiers will be secured.

   The data will be stored in a locked room for 7 years after the final data is collected. After this point, the Data Manager and Dr. Fucito will oversee the process in which data is destroyed or anonymized.

6. If appropriate, has a Certificate of Confidentiality been obtained?

   A COC application is pending.

Section V: Potential benefits

Potential Benefits: Identify any benefits that may be reasonably expected to result from the research, either to the subject(s) or to society at large. (Payment of subjects is not considered a benefit in this context of the risk benefit assessment.)

All participants in this study will receive brief evidence-based advice that may help them improve their sleep and their drinking. Many participants will also engage in sleep self-monitoring that may help participants learn more about their sleep and sleep-related behaviors. Further, many participants will also receive personalized feedback about their sleep and alcohol use that may further increase behavior awareness and promote behavior change. All participants will be offered honorariums for their participation. They will be able to withdraw from the study at any time. There is a need to improve sleep and reduce heavy alcohol consumption and alcohol-related risks among young adults. The purpose of this study is to test a mobile sleep intervention for heavy-drinking young adults. Sleep may be an important gateway topic for intervening on alcohol use in this population. This study may help to engage more heavy-drinking young adults into treatment and reduce the substantial harms and healthcare costs associated with both poor sleep and heavy alcohol use.

SECTION VI: RESEARCH ALTERNATIVES AND ECONOMIC CONSIDERATIONS

1. **Alternatives:** What other alternatives are available to the study subjects outside of the research?

   Alternatives to treatment in this study include evidence-based web-based treatment programs for sleep and/or alcohol use, some of which are available at a cost to the consumer.

2. **Payments for Participation (Economic Considerations):**

   Describe any payments that will be made to subjects, the amount and schedule of payments, and the conditions for receiving this compensation.

   Participants will be paid $30 for completing the intake, $20 each for the week 1 and 2 in-person treatment visits, and $30 each for completing the 3 follow-up visits (i.e., Weeks 4,8, and 12) for a total of $160. Participants will also be compensated for at-home monitoring activities: (1) $2 per day for wearing the Fitbit and Milo Sensor alcohol tracker (14 possible days for a total of $28), (2) $20 for returning the Fitbit and Milo Sensor (2 possible occasions to return for a total of $40), (3) and $1 for completing each sleep diary (14 possible days for a total of $14). The total possible compensation for at-home monitoring is $82. Therefore, the total possible compensation for participants is $242.

3. **Costs for Participation (Economic Considerations):** Clearly describe the subject's costs associated with participation in the research, and the interventions or procedures of the study that will be provided at no cost to subjects.

   Participants will not be charged for any aspects of the treatment. Participants will be randomized to 1 of 3 mobile sleep intervention conditions that will be provided to them at no cost.

4. **In Case of Injury:** This section is required for any research involving more than minimal risk, and for minimal risk research that presents the potential for physical harm (e.g., research involving blood draws).

   Not applicable

a. Will medical treatment be available if research-related injury occurs?
b. Where and from whom may treatment be obtained?
c. Are there any limits to the treatment being provided?
d. Who will pay for this treatment?
e. How will the medical treatment be accessed by subjects?

IMPORTANT REMINDERS

Will this study have a billable service? **Yes** ☐ **No**☒

A billable service is defined as any service rendered to a study subject that, if he/she was not on a study, would normally generate a bill from either Yale-New Haven Hospital or Yale Medical Group to the patient or the patient's insurer. The service may or may not be performed by the research staff on your study, but may be provided by professionals within either Yale-New Haven Hospital or Yale Medical Group (examples include x-rays, MRIs, CT scans, specimens sent to central labs, or specimens sent to pathology). Notes: 1. There is no distinction made whether the service is paid for by the subject or their insurance (Standard of Care) or by the study's funding mechanism (Research Sponsored). 2. This generally includes new services or orders placed in EPIC for research subjects.

If answered, "yes", this study will need to be set up in OnCore, Yale's clinical research management system, for Epic to appropriately route research related charges. Please contact oncore.support@yale.edu

Are there any procedures involved in this protocol that will be performed at YNHH or one of its affiliated entities? **Yes** ☐ **No** ☒

If Yes, please answer questions a through c and note instructions below.
a. Does your YNHH privilege delineation currently include the **specific procedure** that you will perform? **Yes** ☐ **No** ☐
b. Will you be using any new equipment or equipment that you have not used in the past for this procedure? **Yes** ☐ **No** ☐
c. Will a novel approach using existing equipment be applied? **Yes** ☐ **No** ☐

If you answered "no" to question 4a, or "yes" to question 4b or c, please contact the YNHH Department of Physician Services (688-2615) for prior approval before commencing with your research protocol.

IMPORTANT REMINDER ABOUT RESEARCH AT YNHH

Please note that if this protocol includes Yale-New Haven Hospital patients, including patients at the HRU, the Principal Investigator and any co-investigators who are physicians or mid-level practitioners (includes PAs, APRNs, psychologists and speech pathologists) who may have direct patient contact with patients on YNHH premises must have medical staff appointment and

appropriate clinical privileges at YNHH. If you are uncertain whether the study personnel meet the criteria, please telephone the Physician Services Department at 203-688-2615. **By submitting this protocol as a PI, you attest that you and any co-investigator who may have patient contact has a medical staff appointment and appropriate clinical privileges at YNHH.**

BIBLIOGRAPHY & REFERENCES CITED

1. Carey KB, Scott-Sheldon LAJ, Carey MP, DeMartini KS**.** Individual-level interventions to reduce college student drinking: A meta-analytic review. Addictive Behaviors. 2007;32(11):2469-94.
2. Carey KB, Scott-Sheldon LAJ, Elliott JC, Bolles JR, Carey MP**.** Computer-delivered interventions to reduce college student drinking: A meta-analysis. Addiction. 2009;104(11):1807-19.
3. Cronce JM, Larimer ME**.** Individual-focused approaches to the prevention of college student drinking. Alcohol Res Health. 2011;34(2):210-21.
4. Breslau N, Roth T, Rosenthal L, Andreski P**.** Sleep disturbance and psychiatric disorders: a longitudinal epidemiological study of young adults. Biol Psychiatry. 1996;39(6):411-8.
5. Hasler BP, Kirisci L, Clark DB**.** Restless Sleep and Variable Sleep Timing During Late Childhood Accelerate the Onset of Alcohol and Other Drug Involvement. J Stud Alcohol Drugs. 2016;77(4):649-55.
6. Wong MM, Robertson GC, Dyson RB**.** Prospective Relationship Between Poor Sleep and Substance-Related Problems in a National Sample of Adolescents. Alcohol Clin Exp Res. 2015.
7. DeMartini KS, Fucito LM**.** Variations in Sleep Characteristics and Sleep-Related Impairment in At-Risk College Drinkers: A Latent Profile Analysis. Health Psychology. 2014;33(10):1164-73.
8. Singleton Jr RA, Wolfson AR**.** Alcohol consumption, sleep, and academic performance among college students. Journal of Studies on Alcohol and Drugs. 2009;70(3):355-63.
9. Hasler BP, Sitnick SL, Shaw DS, Forbes EE**.** An altered neural response to reward may contribute to alcohol problems among late adolescents with an evening chronotype. Psychiatry Research-Neuroimaging. 2013;214(3):357-64.
10. Hasler BP, Soehner AM, Clark DB**.** Sleep and circadian contributions to adolescent alcohol use disorder. Alcohol. 2015;49(4):377-87.
11. Holm SM, Forbes EE, Ryan ND, Phillips ML, Tarr JA, Dahl RE**.** Reward-related brain function and sleep in pre/early pubertal and mid/late pubertal adolescents. J Adolesc Health. 2009;45(4):326-34.
12. Telzer EH, Fuligni AJ, Lieberman MD, Galvan A**.** The effects of poor quality sleep on brain function and risk taking in adolescence. Neuroimage. 2013;71:275-83.
13. Stepanski EJ, Wyatt JK**.** Use of sleep hygiene in the treatment of insomnia. Sleep Med Rev. 2003;7(3):215-25.
14. Falk D, Yi HY, Hiller-Sturmhofel S**.** An epidemiologic analysis of co-occurring alcohol and drug use and disorders: findings from the National Epidemiologic Survey of Alcohol and Related Conditions (NESARC). Alcohol Res Health. 2008;31(2):100-10.
15. NIAAA**.** Alcohol involvement over the life course. In Chapter 1: Drinking over the lifespan: issues of biology, behavior, and risk. The 10th Special Report to the U.S. Congress on Alcohol and Health. Bethesda, MD; 2000:28-53.
16. NIAAA**.** Alcohol and Other Drugs. Alcohol Alert. 2008;76.

17. Hingson RW, Zha W, Weitzman ER. Magnitude of and trends in alcohol-related mortality and morbidity among U.S. college students ages 18-24, 1998-2005. J Stud Alcohol Drugs Suppl. 2009(16):12-20.
18. Buscemi J, Murphy JG, Martens MP, McDevitt-Murphy ME, Dennhardt AA, Skidmore JR. Help-seeking for alcohol-related problems in college students: correlates and preferred resources. Psychol Addict Behav. 2010;24(4):571-80.
19. Black DR, Coster DC. Interest in a stepped approach model (SAM): identification of recruitment strategies for university alcohol programs. Health Educ Q. 1996;23(1):98-114.
20. Carskadon MA. The second decade. In: Guilleminault C, ed. Sleeping and Waking Disorders: Indications and Techniques. Menlo Park, CA: Addison-Wesley; 1982:99-125.
21. Carskadon MA, Vieira C, Acebo C. Association between Puberty and Delayed Phase Preference. Sleep. 1993;16(3):258-62.
22. Wolfson AR, Carskadon MA. Sleep schedules and daytime functioning in adolescents. Child Development. 1998;69(4):875-87.
23. Wolfson AR. Sleeping patterns of children and adolescents - Developmental trends, disruptions, and adaptations. Child and Adolescent Psychiatric Clinics of North America. 1996;5(3):549-&.
24. Miller MB, DiBello AM, Lust SA, Carey MP, Carey KB. Adequate sleep moderates the prospective association between alcohol use and consequences. Addict Behav. 2016;63:23-8.
25. Mullin BC, Phillips ML, Siegle GJ, Buysse DJ, Forbes EE, Franzen PL. Sleep deprivation amplifies striatal activation to monetary reward. Psychol Med. 2013;43(10):2215-25.
26. Venkatraman V, Chuah YM, Huettel SA, Chee MW. Sleep deprivation elevates expectation of gains and attenuates response to losses following risky decisions. Sleep. 2007;30(5):603-9.
27. Venkatraman V, Huettel SA, Chuah LY, Payne JW, Chee MW. Sleep deprivation biases the neural mechanisms underlying economic preferences. J Neurosci. 2011;31(10):3712-8.
28. Arnedt JT, Conroy DA, Armitage R, Brower KJ. Cognitive-behavioral therapy for insomnia in alcohol dependent patients: a randomized controlled pilot trial. Behav Res Ther. 2011;49(4):227-33.
29. Arnedt JT, Conroy D, Rutt J, Aloia MS, Brower KJ, Armitage R. An open trial of cognitive-behavioral treatment for insomnia comorbid with alcohol dependence. Sleep Med. 2007;8(2):176-80.
30. Brower KJ. Alcohol's effects on sleep in alcoholics. Alcohol Res Health. 2001;25(2):110-25.
31. Brower KJ, Aldrich MS, Hall JM. Polysomnographic and subjective sleep predictors of alcoholic relapse. Alcohol Clin Exp Res. 1998;22(8):1864-71.
32. Brower KJ, Aldrich MS, Robinson EA, Zucker RA, Greden JF. Insomnia, self-medication, and relapse to alcoholism. Am J Psychiatry. 2001;158(3):399-404.
33. Drummond SP, Gillin JC, Smith TL, DeModena A. The sleep of abstinent pure primary alcoholic patients: natural course and relationship to relapse. Alcohol Clin Exp Res. 1998;22(8):1796-802.
34. Brooks AT, Wallen GR. Sleep Disturbances in Individuals with Alcohol-Related Disorders: A Review of Cognitive-Behavioral Therapy for Insomnia (CBT-I) and Associated Non-Pharmacological Therapies. Subst Abuse. 2014;8:55-62.
35. Fucito LM, DeMartini KS, Hanrahan TH, Whittemore R, Yaggi HK, Redeker NS. Perceptions of Heavy-Drinking College Students About a Sleep and Alcohol Health Intervention. Behav Sleep Med. 2015;13(5):395-411.
36. Brown FC, Buboltz Jr WC, Soper B. Relationship of sleep hygiene awareness, sleep hygiene practices, and sleep quality in university students. Behavioral Medicine. 2002;28(1):33-8.

37. Kloss JD, Nash CO, Horsey SE, Taylor DJ. The delivery of behavioral sleep medicine to college students. Journal of Adolescent Health. 2011;48(6):553-61.
38. Mairs L, Mullan B. Self-Monitoring vs. Implementation Intentions: a Comparison of Behaviour Change Techniques to Improve Sleep Hygiene and Sleep Outcomes in Students. Int J Behav Med. 2015;22(5):635-44.
39. Miller WR, Wilbourne PL. Mesa Grande: a methodological analysis of clinical trials of treatments for alcohol use disorders. Addiction. 2002;97(3):265-77.
40. Todd J, Mullan B. The role of self-monitoring and response inhibition in improving sleep behaviours. Int J Behav Med. 2014;21(3):470-7.
41. Helzer JE, Badger GJ, Rose GL, Mongeon JA, Searles JS. Decline in alcohol consumption during two years of daily reporting. Journal of Studies on Alcohol. 2002;63(5):551-8.
42. Quinn JM, Pascoe A, Wood W, Neal DT. Can't Control Yourself? Monitor Those Bad Habits. Personality and Social Psychology Bulletin. 2010;36(4):499-511.
43. Webb TL, Sniehotta FF, Michie S. Using theories of behaviour change to inform interventions for addictive behaviours. Addiction. 2010;105(11):1879-92.
44. Glanz K, Bishop DB. The Role of Behavioral Science Theory in Development and Implementation of Public Health Interventions. Annual Review of Public Health, Vol 31. 2010;31:399-418.
45. Ajzen I, Manstead ASR. Changing health-related behaviours An approach based on the theory of planned behaviour. Scope of Social Psychology: Theory and Applications. 2007:43-63.
46. Leeman RF, Perez E, Nogueira C, DeMartini KS. Very-Brief, Web-Based Interventions for Reducing Alcohol Use and Related Problems among College Students: A Review. Front Psychiatry. 2015;6:129.
47. Trockel M, Manber R, Chang V, Thurston A, Taylor CB. An e-mail delivered CBT for sleep-health program for college students: effects on sleep quality and depression symptoms. J Clin Sleep Med. 2011;7(3):276-81.
48. Demartini KS, Carey KB. Correlates of AUDIT risk status for male and female college students. Journal of American College Health. 2009;58(3):233-9.
49. Anderson M. Technology Device Ownership - 2015. Pew Research Center; 2015.
50. Peters EN, Leeman RF, Fucito LM, Toll BA, Corbin WR, O'Malley SS. Co-occurring marijuana use is associated with medication nonadherence and nonplanning impulsivity in young adult heavy drinkers. Addict Behav. 2012;37(4):420-6.
51. Sobell LC, Agrawal S, Sobell MB, Leo GI, Young LJ, Cunningham JA, et al. Comparison of a quick drinking screen with the timeline followback for individuals with alcohol problems. J Stud Alcohol. 2003;64(6):858-61.
52. Montgomery-Downs HE, Insana SP, Bond JA. Movement toward a novel activity monitoring device. Sleep Breath. 2012;16(3):913-7.
53. de Zambotti M, Baker FC, Willoughby AR, Godino JG, Wing D, Patrick K, et al. Measures of sleep and cardiac functioning during sleep using a multi-sensory commercially-available wristband in adolescents. Physiology & Behavior. 2016;158:143-9.
54. Osterbauer B, Koempel JA, Davidson Ward SL, Fisher LM, Don DM. A comparison study of the Fitbit activity monitor and PSG for assessing sleep patterns and movement in children. Journal of Otolaryngology Advances. 2016;1:24-35.
55. Littner M. Practice parameters for the role of actigraphy in the study of sleep and circadian rhythms: an update for 2002. Sleep. 2003;26(3):337.

56. Kosmadopoulos A, Sargent C, Darwent D, Zhou X, Roach GD. Alternatives to polysomnography (PSG): a validation of wrist actigraphy and a partial-PSG system. Behav Res Methods. 2014;46(4):1032-41.
57. Berger AM, Wielgus KK, Young-McCaughan S, Fischer P, Farr L, Lee KA. Methodological challenges when using actigraphy in research. Journal of Pain and Symptom Management. 2008;36(2):191-9.
58. Kuntsche E, Gmel G. Alcohol consumption in late adolescence and early adulthood - where is the problem? Swiss Medical Weekly. 2013;143.
59. Ebrahim IO, Shapiro CM, Williams AJ, Fenwick PB. Alcohol and sleep I: effects on normal sleep. Alcohol Clin Exp Res. 2013;37(4):539-49.
60. Roehrs T, Roth T. Sleep, Sleepiness, and Alcohol Use. Alcohol Research & Health. 2001;25(2):101.
61. Structured Clinical Interview For DSM-V-RV Axis I Disorders Research Version. New York: American Psychiatric Publishing, Inc; 2014.
62. Read JP, Merrill JE, Kahler CW, Strong DR. Predicting functional outcomes among college drinkers: reliability and predictive validity of the Young Adult Alcohol Consequences Questionnaire. Addict Behav. 2007;32(11):2597-610.
63. Yu L, Buysse DJ, Germain A, Moul DE, Stover A, Dodds NE, et al. Development of short forms from the PROMIS sleep disturbance and Sleep-Related Impairment item banks. Behav Sleep Med. 2011;10(1):6-24.
64. Curran SL, Andrykowski MA, Studts JL. Short-Form of the Profile of Mood States (Poms-Sf) - Psychometric Information. Psychological Assessment. 1995;7(1):80-3.
65. Zavada A, Gordijn MC, Beersma DG, Daan S, Roenneberg T. Comparison of the Munich Chronotype Questionnaire with the Horne-Ostberg's Morningness-Eveningness Score. Chronobiol Int. 2005;22(2):267-78.
66. Monk TH, Reynolds CF, Kupfer DJ, Buysse DJ, Coble PA, Hayes AJ, et al. The Pittsburgh Sleep Diary. J Sleep Res. 1994;3(2):111-20.
67. Demos KE, Hart CN, Sweet LH, Mailloux KA, Trautvetter J, Williams SE, et al. Partial sleep deprivation impacts impulsive action but not impulsive decision-making. Physiology & Behavior. 2016;164:214-9.
68. Ahmadi A, Pearlson GD, Meda SA, Dager A, Potenza MN, Rosen R, et al. Influence of Alcohol Use on Neural Response to Go/No-Go Task in College Drinkers. Neuropsychopharmacology. 2013;38(11):2197-208.
69. Valeri L, Vanderweele TJ. Mediation analysis allowing for exposure-mediator interactions and causal interpretation: theoretical assumptions and implementation with SAS and SPSS macros. Psychol Methods. 2013;18(2):137-50.
