# Supplementary material for: Wearable Intervention for Alcohol Use Risk and Sleep in Young Adults: A Randomized Clinical Trial
Source: JAMA Netw Open. 2025 May 30;8(5):e2513167. doi: 10.1001/jamanetworkopen.2025.13167 (PMC12125640; doi:10.1001/jamanetworkopen.2025.13167)
Supplement: Supplement 2. — eFigure 1. Sample Smartphone Diary Image eFigure 2. Sample Participant Feedback [file jamanetwopen-e2513167-s002.pdf]

Supplementary Online Content

Fucito LM, Ash GI, Wu R, et al. Wearable intervention for drinking risk and sleep in young adults: a randomized clinical trial. *JAMA Netw Open.* 2025;8(5):e2513167. doi:10.1001/jamanetworkopen.2025.13167

eFigure 1. Sample Smartphone Diary Image

eFigure 2. Sample Participant Feedback

This supplementary material has been provided by the authors to give readers additional information about their work.

eFigure 1. Sample Smartphone Diary Image

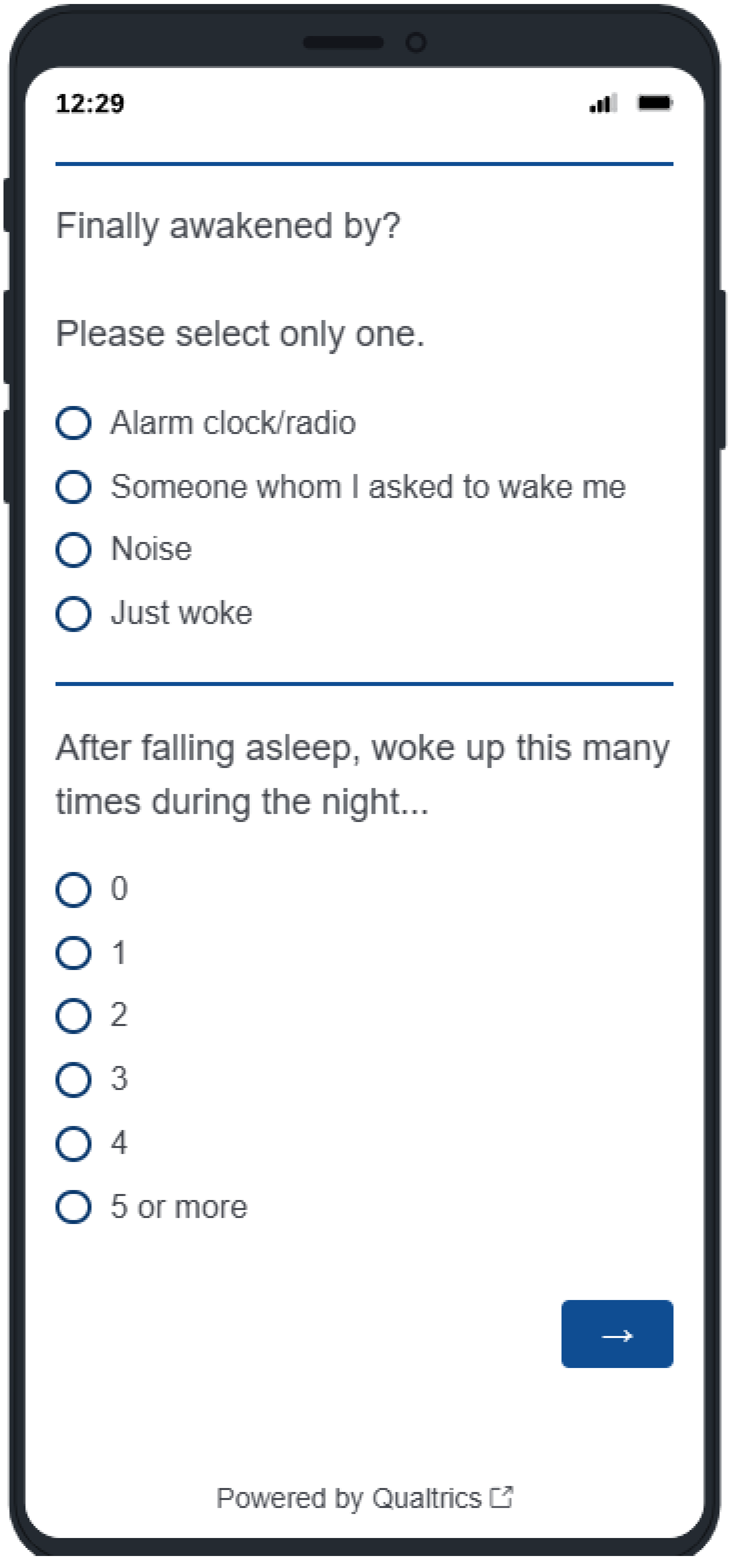

© 2025 Fucito LM et al. *JAMA Network Open*.

eFigure 2. Sample Participant Feedback

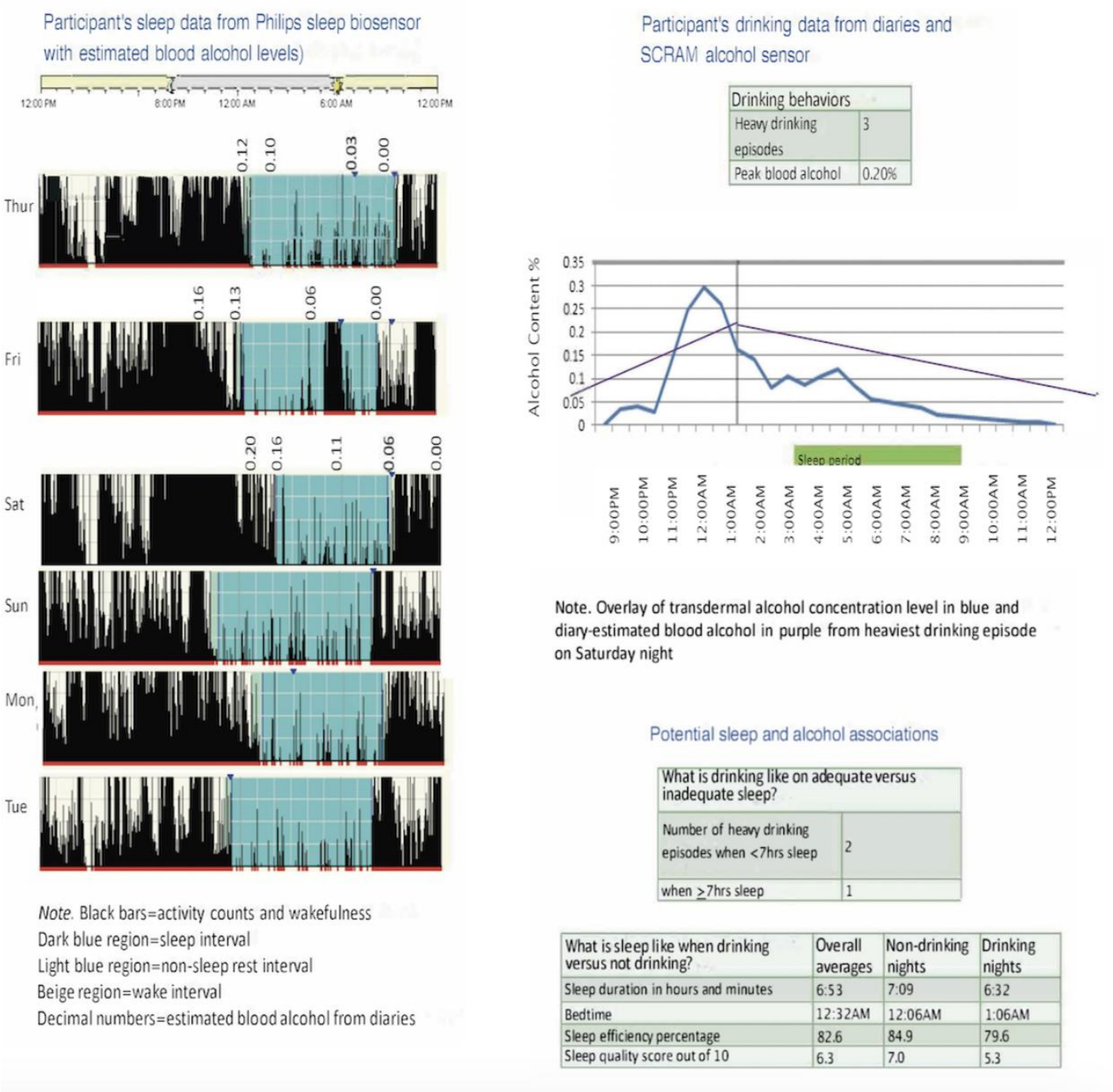

Figure previously published and copyrighted by the authors.

Fucito, L.M., et al., A Multimodal Mobile Sleep Intervention for Young Adults Engaged in Risky Drinking: Protocol for a Randomised Controlled Trial. *JMIR Research Protocols,* 2021. 10(2): e26557.

A link to the original publication can be found here: https://www.researchprotocols.org/2021/2/e26557/

© 2025 Fucito LM et al. *JAMA Network Open*.

© 2025 Fucito LM et al. *JAMA Network Open*.
