# Supplementary material for: Wearable Intervention for Alcohol Use Risk and Sleep in Young Adults: A Randomized Clinical Trial
Source: JAMA Netw Open. 2025 May 30;8(5):e2513167. doi: 10.1001/jamanetworkopen.2025.13167 (PMC12125640; doi:10.1001/jamanetworkopen.2025.13167)
Supplement: Supplement 3. — Data Sharing Statement [file jamanetwopen-e2513167-s003.pdf]

Data Sharing Statement

Fucito. Wearable Intervention for Alcohol Use Risk and Sleep in Young Adults. *JAMA Netw Open*. Published May 30, 2025. doi:10.1001/jamanetworkopen.2025.13167

Data
Additional Information: clinicaltrials.gov #NCT03658954, https://clinicaltrials.gov/study/NCT03658954
Data available: Yes
Data types: Deidentified participant data
How to access data: Individuals can access data by contacting Dr. Fucito, the principal investigator.
When available: With publication

Supporting Documents
Document types: None

Additional Information
Who can access the data: Data will be made available beginning 9 months after publication of the primary trial publication upon request by researchers who agree to complete a signed data access agreement by contacting the corresponding author and principal investigator, Dr. Fucito.
Types of analyses: Requests by researchers who provide a methodologically sound proposal for any purpose.
Mechanisms of data availability: After approval of a proposal and with a signed data access agreement.
